# Association Between State Opioid Prescribing Cap Laws and Receipt of Opioid Prescriptions Among Children and Adolescents

**DOI:** 10.1001/jamahealthforum.2022.2461

**Published:** 2022-08-05

**Authors:** Elizabeth M. Stone, Kayla N. Tormohlen, Alexander D. McCourt, Ian Schmid, Elizabeth A. Stuart, Corey S. Davis, Mark C. Bicket, Emma E. McGinty

**Affiliations:** 1 Department of Health Policy and Management, Johns Hopkins Bloomberg School of Public Health, Baltimore, Maryland; 2 Department of Mental Health, Johns Hopkins Bloomberg School of Public Health, Baltimore, Maryland; 3 Network for Public Health Law, Los Angeles, California; 4 Department of Anesthesiology, School of Public Health, University of Michigan, Ann Arbor; 5 Opioid Prescribing Engagement Network, Institute for Healthcare Policy and Innovation, University of Michigan, Ann Arbor; 6 Visiting Fellow, OptumLabs, Cambridge, Massachusetts

## Abstract

**Importance:**

High-dose and long-duration opioid prescriptions remain relatively common among children and adolescents, but there is insufficient research on the association of state laws limiting the dose and/or duration of opioid prescriptions (referred to as opioid prescribing cap laws) with opioid prescribing for this group.

**Objective:**

To examine the association between state opioid prescribing cap laws and the receipt of opioid prescriptions among children and adolescents.

**Design, Setting, and Participants:**

This repeated cross-sectional study used a difference-in-differences approach accounting for staggered policy adoption to assess the association of state opioid prescribing cap laws in the US from January 1, 2013, to December 31, 2019, with receipt of opioid prescriptions among children and adolescents. Analyses were conducted between March 22 and December 15, 2021. Data were obtained from the OptumLabs Data Warehouse, a national commercial insurance claims database. The analysis included 482 118 commercially insured children and adolescents aged 0 to 17 years with full calendar-year continuous insurance enrollment between 2013 and 2019. Individuals were included for every year in which they were continuously enrolled; they did not need to be enrolled for the entire 7-year study period. Those with any cancer diagnosis were excluded from analysis.

**Exposure:**

Implementation of a state opioid prescribing cap law between January 1, 2017, and July 1, 2019. This date range allowed analysis of the same number years for both pre-cap and post-cap data.

**Main Outcomes and Measures:**

Outcomes of interest included receipt of any opioid prescription and, among those with at least 1 opioid prescription, the mean number of opioid prescriptions, mean morphine milligram equivalents (MMEs) per day, and mean days’ supply.

**Results:**

Among 482 118 children and adolescents (754 368 person-years of data aggregated to the state-year level), 245 178 (50.9%) were male, with a mean (SD) age of 9.8 (4.8) years at the first year included in the sample (data on race and ethnicity were not collected as part of this data set, which was obtained from insurance billing claims). Overall, 10 659 children and adolescents (2.2%) received at least 1 opioid prescription during the study period. Among those with at least 1 prescription, the mean (SD) number of filled opioid prescriptions was 1.2 (0.8) per person per year. No statistically significant association was found between state opioid prescribing cap laws and any outcome. After opioid prescribing cap laws were implemented, a −0.001 (95% CI, −0.005 to 0.002) percentage point decrease in the proportion of youths receiving any opioid prescription was observed. In addition, percentage point decreases of −0.01 (95% CI, −0.10 to 0.09) in high-dose opioid prescriptions (>50 MMEs per day) and −0.02 (95% CI, −0.12 to 0.08) in long-duration opioid prescriptions (>7 days’ supply) were found after cap laws were implemented.

**Conclusions and Relevance:**

In this cross-sectional study, no association was observed between state opioid prescribing cap laws and the receipt of opioid prescriptions among children and adolescents. Alternative strategies, such as opioid prescribing guidelines tailored to youths, are needed.

## Key Points

**Question:**

What is the association between state laws limiting the dose and/or duration of opioid prescriptions (opioid prescribing cap laws) and the receipt of opioid prescriptions among children and adolescents?

**Findings:**

In this cross-sectional study of 482 118 children and adolescents in the US, a difference-in-differences analysis accounting for staggered policy adoption found no statistically significant association between state opioid prescribing cap laws and the receipt of any opioid prescription or the number, dose, or duration of opioid prescription fills for youths.

**Meaning:**

This study found no association between state opioid prescribing cap laws and the receipt of opioid prescriptions among children and adolescents, suggesting that alternative strategies, such as opioid prescribing guidelines tailored to youths, are needed.

## Teaser

This cross-sectional study assesses the association between state laws limiting the dose and/or duration of opioid prescriptions and the receipt of opioid prescriptions among children and adolescents in the US.

## Introduction

The US opioid crisis continues to be a substantial public health issue among children and adolescents. Between 1999 and 2016, 9000 youths died of opioid overdose, and prescription opioids were associated with almost three-quarters of those deaths.^1^ As is the case for adults, high-dose and long-duration opioid prescriptions for children and adolescents have been associated with increased overdose risk.^2^ Clinical guidelines for opioid prescribing do not include recommendations specific to youths.^3^ Children and adolescents commonly receive opioid prescriptions that meet or exceed high-dose and long-duration thresholds identified as placing adults at high risk of overdose.^2,4^ A recent study by Chua et al^4^ found that 46% of opioid prescriptions for children and young adults in 2019 would have been considered high risk, even for prescribing to adults.

One strategy US states have used in an attempt to address high-dose and long-duration opioid prescribing for people of all ages is the implementation of laws that limit the dose and/or duration of opioid prescriptions for acute pain (referred to as opioid prescribing cap laws). As of December 2019, 39 states had implemented an opioid prescribing cap law. Specifics of the prescribing caps vary across states, with laws limiting the dose (eg, <50 morphine milligram equivalents [MMEs] per day) and/or duration (eg, <7 days’ supply) of an opioid prescription.

All states with opioid prescribing cap laws that limit prescriptions for adults also limit prescriptions for children and adolescents. In certain cases, these limits are stricter for youths. For example, Connecticut limits initial opioid prescriptions for adults to a 7-day supply and all opioid prescriptions for youths to a 5-day supply,^5^ and Alaska has a 7-day prescribing cap limit that applies to all opioid prescriptions for children and adolescents but only to initial prescriptions for adults.^6^

Although 1 previous study^7^ found no association between state opioid prescribing cap laws and opioid poisonings among individuals younger than 20 years, no previous studies have, to our knowledge, examined the association of state opioid prescribing cap laws with opioid prescribing for children and adolescents. Our study aimed to address this gap by examining the association between state opioid prescribing cap laws and the receipt of opioid prescriptions among children and adolescents. We hypothesized that state prescribing cap laws would be associated with reductions in the volume, dose, and duration of filled opioid prescriptions in this population.

## Methods

This study was approved by the institutional review board of the Johns Hopkins Bloomberg School of Public Health and determined to be exempt from informed consent because it was a secondary analysis of existing deidentified data. The study followed the Strengthening the Reporting of Observational Studies in Epidemiology (STROBE) reporting guideline for cross-sectional studies.

### Data

We used deidentified administrative insurance claims data from the OptumLabs Data Warehouse^8^ from January 1, 2013, to December 31, 2019. This data set included information on member coverage and enrollment as well as medical and pharmaceutical claims for commercially insured individuals across all 50 US states and the District of Columbia.^8^

### Identification of Prescribing Cap Laws

States with opioid prescribing cap laws were identified through standard legal mapping techniques that included searches of the Westlaw legal database^9^ and state legislative and regulatory documents to identify the presence of a state opioid prescribing cap law, the law’s implementation date, and the important provisions in each state law.^10^ Further details of the legal mapping process have been reported elsewhere.^11,12^

### Sample

Our study focused on children and adolescents aged 0 to 17 years. Individuals in this age group were included in the sample for each year from January 1, 2013, to December 31, 2019, in which they had a full 12 months of continuous medical and pharmacy enrollment. In other words, if a child had 12 months of continuous insurance enrollment in 2013 but only 6 months of continuous enrollment in 2014, the child was included in the sample for 2013 only. Individuals with any cancer diagnosis were excluded from the analysis. A total of 482 118 individual children and adolescents aged 0 to 17 years were included.

We identified 33 treatment states with opioid prescribing cap laws implemented between January 1, 2017, and July 1, 2019. We coded the year of implementation as the first calendar year in which a state had an opioid prescribing cap law in place for most of the year (ie, before July 1). Using this strategy, we coded 12 states (Connecticut, Delaware, Kentucky, Maryland, Maine, New Hampshire, New Jersey, New York, Pennsylvania, Rhode Island, Utah, and Virginia) as implementing a law in 2017, 12 states (Alaska, Arizona, Colorado, Hawaii, Indiana, Louisiana, North Carolina, Nevada, Ohio, South Carolina, Vermont, and West Virginia) as implementing a law in 2018, and 9 states (Arkansas, Florida, Michigan, Missouri, Mississippi, Nebraska, Oklahoma, Tennessee, and Washington) as implementing a law in 2019. Fifteen states (Alabama, California, Georgia, Iowa, Idaho, Kansas, Minnesota, Montana, North Dakota, New Mexico, Oregon, South Dakota, Texas, Wisconsin, and Wyoming) and the District of Columbia, which did not have a prescribing cap law implemented before July 1, 2019, were included in the comparison group.

The analytic approach used in this study estimated cohort-specific associations based on the policy implementation year. Because this approach can produce unstable estimates when there are fewer than 5 units (ie, states) in a cohort,^13^ 2 states (Illinois and Massachusetts) that implemented an opioid prescribing cap law before 2017 were excluded from this study. All 33 states with prescribing cap laws included limits on prescription duration (ie, number of days’ supply; mode, 7 days; range, 3-30 days). Of the 14 states with laws limiting dose, 7 states included limits on specific doses (range, 24-120 MMEs per day), and the other 7 states set the limit as the lowest effective dose. Fourteen laws applied only to initial opioid prescriptions, and 22 laws included professional judgment exemptions that allowed prescribers to override the limits set forth in the law based on their professional judgment. A full list of states with opioid prescribing cap laws as well as implementation dates and provisions is provided in eTable 1 and eTable 2 in the Supplement.

### Opioid Outcome Measures

A measure of the receipt of any opioid prescription was constructed by first identifying opioid prescriptions at the individual level for each year and then aggregating those prescriptions to the state-year level to measure the proportion of children and adolescents in the state who received any opioid prescription per year. Among individuals who received at least 1 opioid prescription in a given year, we measured the mean number of opioid prescriptions, mean MMEs per day, and mean days’ supply per person per year, then aggregated those data to the state-year level. We also constructed measures identifying the proportion of children and adolescents with at least 1 filled opioid prescription who received more than a 3-day, 5-day, or 7-day supply of opioids and greater than 30, 50, or 90 MMEs per day in a state-year. Opioid prescriptions were identified using the Centers for Disease Control and Prevention opioid and oral MME conversion file.^14^ We excluded opioid agonist medications primarily used to treat opioid use disorder from the analyses.

### Statistical Analysis

Recent literature^15,16^ has reported that traditional difference-in-differences methods with 2-way fixed effects produce biased results in settings in which policy adoption is staggered across units over time. Because this staggered policy adoption occurred across the states in this study, we used an adapted difference-in-differences design developed by Callaway and Sant’Anna.^16^ The Callaway and Sant’Anna^16^ approach estimates the mean treatment effect for each cohort period. The cohorts were defined by year of policy implementation (2017, 2018, or 2019). We used the Callaway and Sant’Anna^16^ method to estimate the mean treatment effect for the treated group. This estimate can be interpreted as the mean difference between outcomes in the presence of an opioid prescribing cap law and outcomes had the law not been implemented within the treatment group, accounting for the length of time since the law was enacted (ie, the change in outcomes associated with the opioid prescribing cap law).

The primary analysis for this study used balanced models, in which each cohort (2017, 2018, and 2019) had the same number of years of both pre–cap law and post–cap law data; this approach minimized bias introduced by changes in treatment and comparison group composition over time.^16^ This process resulted in a study period from 2013 to 2019, with each cohort having 3 years of pre–cap law data and 1 year of post–cap law data. Because there was no evidence of differential pre–study period patterns between treatment and comparison states, the main models did not include covariates. Differences in pre–study period patterns and demographic characteristics were assessed using unpaired 2-tailed *t* tests that compared states with vs without prescribing cap laws.

We conducted 4 sets of sensitivity analyses. First, we repeated the main analysis stratified by 3 age groups: 0 to 5 years, 6 to 11 years, and 12 to 17 years. Second, we estimated cohort-specific associations to examine opioid prescribing cap laws and outcomes at 2 years and 3 years after cap law implementation. Third, we repeated the primary analysis, including covariates for the proportion of female individuals, the mean age, the proportion of individuals with any mental disorder diagnosis, and the proportion of individuals with any substance use disorder diagnosis. Fourth, we repeated the main analysis stratified by type of opioid prescribing cap law (ie, laws that limited duration only, laws that limited both duration and dose, laws that did vs did not apply only to initial prescriptions, laws that did vs did not include a professional judgment exemption, and laws that did vs did not include a surgical pain exemption).

Analyses were conducted between March 22 and December 15, 2021. All analyses were performed using R software, version 4.0.3 (R Foundation for Statistical Computing) using the did package developed by Callaway and Sant’Anna.^17^ The statistical significance threshold was 2-tailed *P* < .05.

## Results

Among 482 118 children and adolescents aged 0 to 17 years, 236 940 (49.1%) were female, and 245 178 (50.9%) were male; the mean (SD) age was 9.8 (4.9) years at the first year included in the sample (data on race and ethnicity were not collected as part of this data set, which was obtained from insurance billing claims). Overall, 10 659 children and adolescents (2.2%) received at least 1 opioid prescription during the study period. Among those with at least 1 prescription, the mean (SD) number of filled opioid prescriptions was 1.2 (0.8) per person per year. Prescriber specialty was associated with 87.2% of the opioid prescription fills across the study period. The most frequent prescriber specialties identified were dental (37.4%), orthopedic (10.4%), and emergency medicine (9.0%).

**Figure 1.**
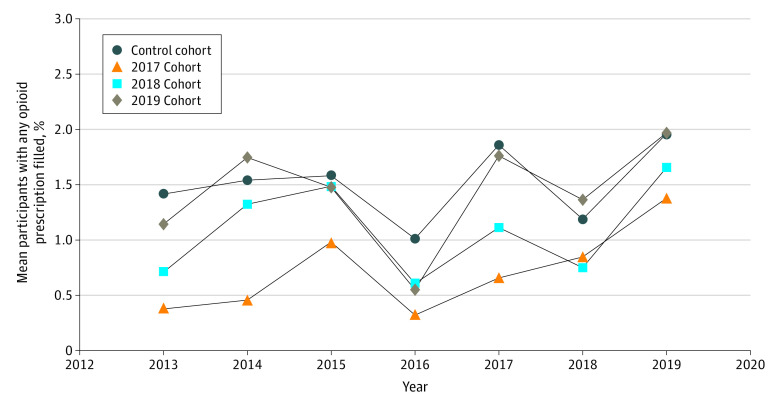
Mean Percentage of Children and Adolescents With Any Filled Opioid Prescription by Treatment Cohort, 2013-2019 Descriptive statistics were derived using insurance claims data from the OptumLabs Data Warehouse for the 12 states that implemented an opioid prescribing cap law in 2017, the 12 states that implemented an opioid prescribing cap law in 2018, the 9 states that implemented an opioid prescribing cap law in 2019, and the 16 states with no opioid prescribing cap law (control cohort).

Individuals were included in the study sample for all calendar years in which they were continuously enrolled, resulting in 754 368 person-years of data aggregated by year for each of the 33 states implementing an opioid prescribing cap law between 2017 and 2019 and each of the 16 states without an opioid prescribing cap law. Over time, population characteristics were generally the same in the total sample and in both the treatment and control cohorts (eTables 18-20 in the Supplement). State-year–level demographic characteristics and filled opioid prescription measures were similar in both the treatment and comparison groups, with 1 exception: comparison group states that did not implement an opioid prescribing cap law during the study period had a higher proportion of individuals receiving at least 1 opioid prescription in the pre–cap law period (1.4%) relative to treatment states that implemented cap laws (0.9%; *P* = .004) (Table 1). The mean percentages of individuals receiving at least 1 opioid prescription in each year of the study period by treatment group are shown in Figure 1.

**Table 1.** State-Year–Level Characteristics of Participants Before Implementation of State Opioid Prescribing Cap Laws, 2013-2016

Characteristic	Participants, %^a^	
	States with an opioid prescribing cap law^b^	States without an opioid prescribing cap law^c^
**State demographic characteristics**		
Sex		
Female	49.3	48.7
Male	50.7	51.3
Age, y		
Mean	10.1	10.2
Group		
0-5	19.4	20.9
6-11	35.6	36.0
12-17	45.1	43.1
Any mental illness	6.4	6.1
Any substance use disorder	0.1	0.1
**Opioid prescriptions**		
Receipt of ≥1 prescription	0.9	1.4^d^
Among those receiving ≥1 prescription^e^		
Total prescriptions, No.	1.0	1.1
Dose, MMEs/d/y		
Mean	32.6	42.2
>30	37.0	44.5
>50	12.3	13.1
>90	1.9	3.6
Duration, days’ supply/y		
Mean	4.6	3.9
>3	49.9	45.5
>5	22.9	16.3
>7	12.7	7.6

Abbreviation: MME, morphine milligram equivalent.

^a^ Because this table shows state year–level characteristics, the percentages do not directly align with individual-level numbers. For example, 49.3% female sex represents the mean of the 33 treatment state means across the 3-year baseline period. Therefore, numerators and denominators for percentages (and SDs for means) could not be accurately reported.

^b^ A total of 33 states implemented opioid prescribing cap laws in 2017, 2018, and 2019. The 2017 cohort included Connecticut, Delaware, Kentucky, Maryland, Maine, New Hampshire, New Jersey, New York, Pennsylvania, Rhode Island, Utah, and Virginia. The 2018 cohort included Alaska, Arizona, Colorado, Hawaii, Indiana, Louisiana, North Carolina, Nevada, Ohio, South Carolina, Vermont, and West Virginia. The 2019 cohort included Arkansas, Florida, Michigan, Missouri, Mississippi, Nebraska, Oklahoma, Tennessee, and Washington.

^c^ A total of 16 states, including Alabama, California, District of Columbia, Georgia, Iowa, Idaho, Kansas, Minnesota, Montana, North Dakota, New Mexico, Oregon, South Dakota, Texas, Wisconsin, and Wyoming, did not implement opioid prescribing cap laws.

^d^ *P* = .004.

^e^ Opioid prescription volume, dose, and duration measures were calculated as mean per person per year.

**Figure 2.**
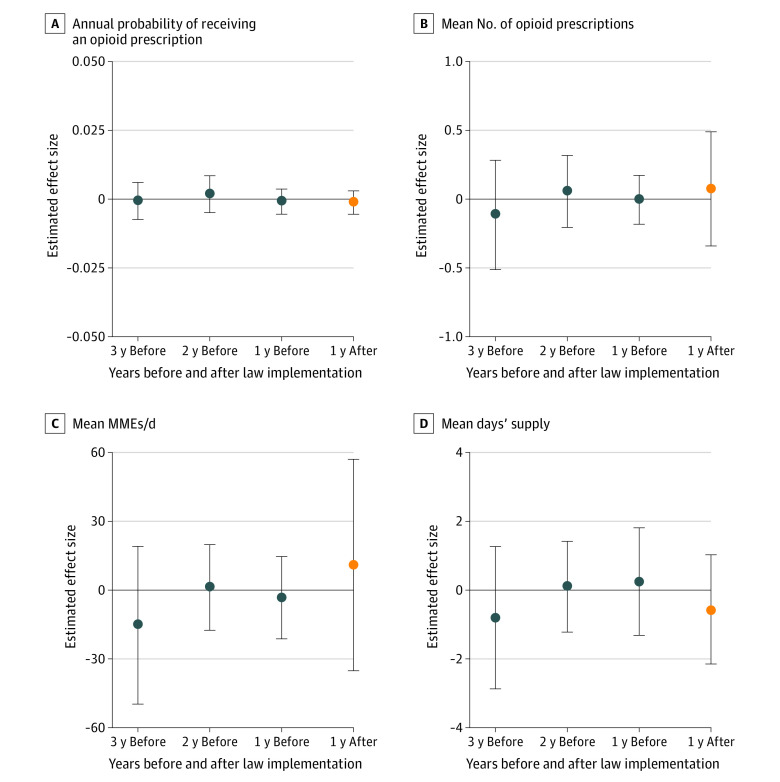
Change in Annual Probability of Receiving an Opioid Prescription and Change in Annual Volume, Dose, and Duration of Opioid Prescriptions per Person per Year Changes associated with state opioid prescribing cap laws during the first year after implementation among those receiving ≥1 opioid prescription. Effect size estimates were derived using the staggered adoption design of Callaway and Sant’Anna.^16^ Insurance claims data were obtained from the OptumLabs Data Warehouse for the 33 states that implemented an opioid prescribing cap law between 2017 and 2019 and the 16 control states with no opioid prescribing cap law. Vertical bars represent 95% CIs. MME indicates morphine milligram equivalent.

No association was found between opioid prescribing cap laws and the proportion of children and adolescents receiving any opioid prescription per year (mean treatment effect, −0.001 [95% CI, −0.005 to 0.002] percentage points) (Figure 2). There was also no significant difference between prescribing cap laws and the mean number of filled opioid prescriptions per person per year (mean treatment effect, 0.08 [95% CI, −0.27 to 0.42] percentage points), mean MMEs per day per person per year (mean treatment effect, 11.16 [95% CI, −29.74 to 52.06] percentage points), or mean days’ supply per person per year (mean treatment effect, −0.54 [95% CI, −2.02 to 0.94] percentage points) (Figure 2). Changes in outcomes after implementation of opioid prescribing cap laws were nonsignificant with regard to the proportions of youths receiving opioid prescriptions at any level of dose or duration (Table 2). For example, high-dose opioid prescriptions (>50 MMEs per day) decreased by −0.01 (95% CI, −0.10 to 0.09) percentage points, and long-duration opioid prescriptions (>7 days’ supply) decreased by −0.02 (95% CI, −0.12 to 0.08) percentage points, but these changes were not statistically significant.

**Table 2.** Change in Proportion of Children and Adolescents per Year Receiving Opioid Prescriptions at Specified Dose or Duration Levels^a^

Outcome	Estimated effect size (95% CI)
Dose, MMEs/d/y	
>30	0.01 (−0.05 to 0.28)
>50	−0.01 (−0.10 to 0.09)
>90	0.04 (−0.03 to 0.12)
Duration, days’ supply/y	
>3	−0.08 (−0.25 to 0.09)
>5	−0.04 (−0.17 to 0.09)
>7	−0.02 (−0.12 to 0.08)

Abbreviation: MME, morphine milligram equivalent.

^a^ Among those receiving 1 or more opioid prescriptions associated with state opioid prescribing cap laws during the first year after implementation. Effect estimates were obtained using the staggered adoption design of Callaway and Sant’Anna^16^; insurance claims data were obtained from the OptumLabs Data Warehouse for the 33 states that implemented an opioid prescribing cap law between 2017 and 2019 and the 16 control states that did not implement an opioid prescribing cap law.

**Figure 3.**
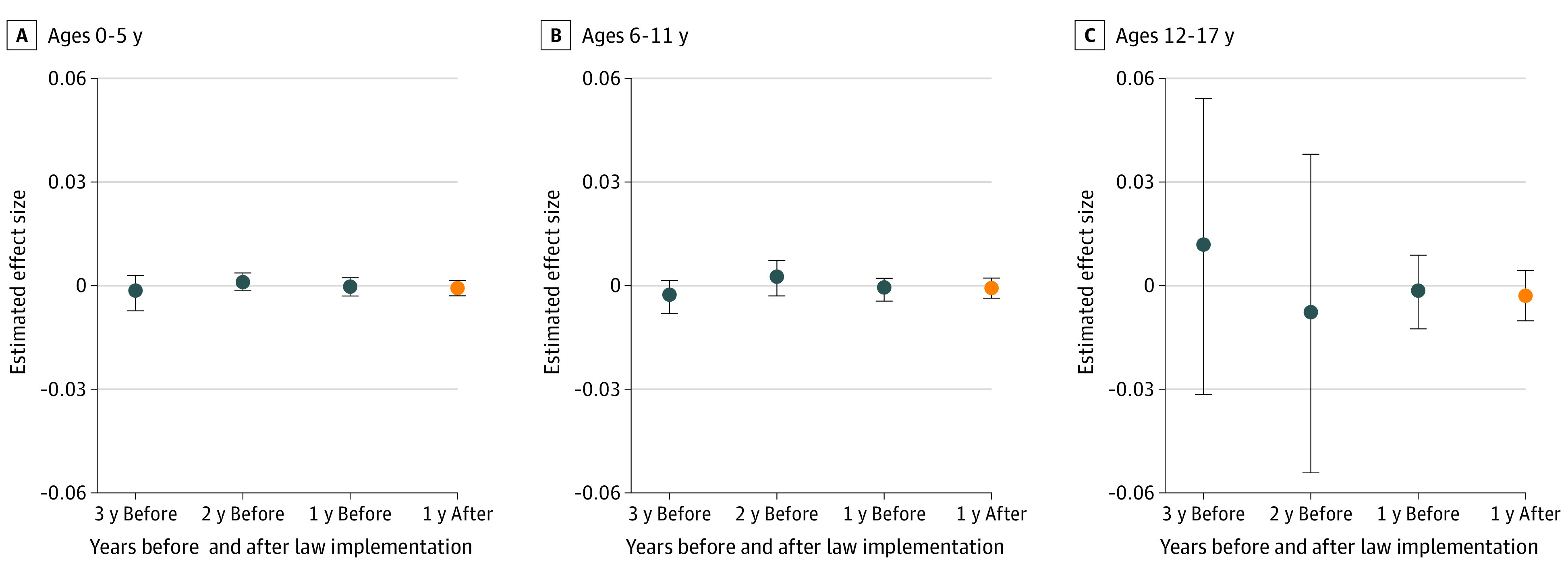
Change in Annual Probability of Receiving Any Opioid Prescription Among Children and Adolescents Changes associated with state opioid prescribing cap laws during the first year after implementation. Effect size estimates were derived using the staggered adoption design of Callaway and Sant’Anna.^16^ Insurance claims data were obtained from the OptumLabs Data Warehouse for the 33 states that implemented an opioid prescribing cap law between 2017 and 2019 and the 16 control states with no opioid prescribing cap law. Vertical bars represent 95% CIs.

No association was found between opioid prescribing cap laws and the proportion of children and adolescents receiving any opioid prescription per year for any of the 3 age groups (mean treatment effect for ages 0-5 years: −0.001 [95% CI, −0.003 to 0.002] percentage points; ages 6-11 years: −0.001 [95% CI, −0.003 to 0.002] percentage points; ages 12-17 years: −0.003 [95% CI, −0.010 to 0.004] percentage points) (Figure 3). In addition, no significant changes after implementation of cap laws were found with regard to the mean number of opioid prescription fills (mean treatment effect for ages 0-5 years: 0.024 [95% CI, −0.306 to 0.354] percentage points; ages 6-11 years: −0.088 [95% CI, −0.347 to 0.170] percentage points; ages 12-17 years: 0.117 [95% CI, −0.274 to 0.508] percentage points), the mean MMEs per day (mean treatment effect for ages 0-5 years: −1.050 [95% CI, −4.260 to 2.160] percentage points; ages 6-11 years: 0.728 [95% CI, −9.376 to 10.832] percentage points; ages 12-17 years: 10.887 [95% CI, −35.116 to 56.891] percentage points), the mean days’ supply (mean treatment effect for ages 0-5 years: −0.639 [95% CI, −3.979 to 2.701] percentage points; ages 6-11 years: −1.192 [95% CI, −2.820 to 0.435] percentage points; ages 12-17 years: −0.088 [95% CI, −1.582 to 1.406] percentage points), or the proportions of individuals filling opioid prescriptions at any level of dose (eg, mean treatment effect for >30 MMEs per day for ages 0-5 years: −0.013 [95% CI, −0.029 to 0.003] percentage points; ages 6-11 years: 0.018 [95% CI, −0.067 to 0.103] percentage points; ages 12-17 years: 0.127 [95% CI, −0.046 to 0.299] percentage points) or duration (eg, mean treatment effect for >3 days’ supply per year for ages 0-5 years: −0.063 [95% CI, −0.301 to 0.174] percentage points; ages 6-11 years: −0.157 [95% CI, −0.345 to 0.031] percentage points; ages 12-17 years: −0.051 [95% CI, −0.226 to 0.123] percentage points) in any of the 3 age groups (eTables 3-5 in the Supplement).

Results of other sensitivity analyses were also consistent with the main findings. We did not observe significant changes in outcomes associated with prescribing cap laws with regard to filled prescription outcomes at 2 years or 3 years after cap law implementation (eTables 6-8 in the Supplement). Results were similar when adjusting models for covariates (eTable 9 in the Supplement) and when stratifying the sample by type of prescribing cap law (eTables 10-17 in the Supplement).

## Discussion

In this cross-sectional study involving a national sample of commercially insured children and adolescents, we found no association between state opioid prescribing cap laws and the receipt of opioid prescriptions among children and adolescents. To our knowledge, this study was the first to assess the association of opioid prescribing cap laws with the receipt of opioid prescriptions among youths using a staggered difference-in-differences approach to address challenges pertaining to effect measurement when laws are implemented in different states at different times. Findings of no change in filled prescription outcomes after the implementation of opioid prescribing cap laws remained consistent across all sensitivity analyses, including analyses across 3 age groups. This study’s findings were consistent with those of a recent study^7^ of National Poison System data from 2005 to 2017 that found no association between state opioid prescribing cap laws and opioid-related poisonings among children and young adults. Null findings were also consistent with recent studies^18,19,20^ reporting little or no association between state opioid prescribing cap laws and opioid prescription receipt or opioid overdose among adults.

Difficulty in implementing and enforcing state opioid prescribing cap laws may have implications for filled prescription outcomes.^21^ Exemptions, particularly those for professional judgment, and lack of state capacity to monitor adherence may inhibit the intended outcome of decreasing high-dose and long-duration opioid prescriptions.^21^ For example, in many states, a medical licensing board might only look at a prescriber’s adherence to a prescribing cap law in response to a complaint, with little to no active enforcement of the policy.^21^

In this analysis, receipt of an opioid prescription was relatively rare. This finding was consistent with that of another study^4^ reporting that privately insured children and adolescents were less likely to fill an opioid prescription than those with Medicaid or no insurance. Overall, opioid prescribing for youths has decreased over the past decade,^4,22,23^ and we found no change in opioid prescription fills, volume, or dose associated with state prescribing cap laws. However, previous studies have found that receipt of long-duration opioid prescriptions with 30 or more days’ supply increased among children and adolescents from 2006 to 2018,^22,23^ and the mean amount of opioids dispensed and the rate of high-dose prescriptions for young children aged 0 to 5 years increased over the same period.^22^ In 2018, 1 in 5 opioid prescriptions for children aged 0 to 5 years was classified as a high-dose prescription.^22^ Approximately 20% of adolescents and young adults reported misusing opioids, and two-thirds of individuals with an opioid use disorder first used opioids before age 25 years.^24^ There are currently no guidelines on opioid prescribing for those younger than 18 years. Recent evidence^4,25^ suggests that even relatively short-duration and low-dose opioid prescriptions can increase the risk of long-term opioid use among children and young adults and that medications such as ibuprofen provide equivalent pain management for medical procedures common among children and adolescents, such as tooth extraction and tonsillectomy.

To address the ongoing opioid crisis successfully, specific focus on children and adolescents is needed. Development of guidelines pertaining to opioid prescribing for youths could be targeted toward health care professionals who frequently prescribe opioids to this population (eg, dentists or orthopedic specialists). The lack of targeted guidance for children and adolescents may partially explain the null findings in this study because most filled prescriptions for youths were within limits (eg, <7 days’ supply) set forth by state opioid prescribing laws. Future guidelines could provide more specific information about what constitutes high-risk or appropriate prescribing for children and adolescents (eg, <3 days’ supply). Creation of clinical prescribing guidelines for youths may help to reduce opioid prescribing for this population while ensuring the adequate treatment of pain.

### Limitations

This study has several limitations. Although weight-based dosing for opioid prescription was not possible because claims data lack information on weight, no differences were present in older subgroups, for whom most opioid prescriptions are based on weight-independent dosing (eg, similar dosing for weight >50 kg). Although approximately 50% of children and adolescents in the US are covered by private employer-based insurance, the inclusion of commercially insured individuals in this study limits generalizability to other populations (eg, Medicaid-insured or uninsured children and adolescents).^26^ The use of insurance claims data also limits the ability to identify prescriptions not filled or covered by insurance and does not necessarily equate to consumption of the opioids prescribed.

For the current study sample, we required calendar-year continuous enrollment, resulting in a changing case mix across study years. We expect the consequences of this change for the study results were minimal because the population characteristics remained largely the same over time in the sample as a whole and in both the treatment and control groups (eTables 18-20 in the Supplement). This continuous enrollment requirement may also limit the generalizability of findings to populations with lower income who may experience more frequent changes in insurance coverage and therefore would be excluded from this sample. Because the analysis is at the state level, and there are a limited number of states, it may be underpowered to detect a difference in outcomes associated with opioid prescribing cap laws. However, point estimates are close to zero for most analyses.

Opioid prescribing cap laws have been implemented fairly recently, limiting potential observation of outcomes. However, sensitivity analyses did not suggest that there were changes associated with cap law policy either 2 years or 3 years after policy implementation. States have implemented many policies attempting to limit high-risk opioid prescribing practices. We did not control for other opioid laws in this analysis, although there is evidence that other policies (eg, prescription drug monitoring program requirements or pain management clinic regulations) have had no association with the receipt of opioid prescriptions in adult populations.^27,28^

## Conclusion

In this repeated cross-sectional study, state opioid prescribing cap laws were not associated with any change in the receipt of opioid prescriptions among children and adolescents. Guidance on prescribing for this age group may be needed as part of the response to the ongoing opioid crisis.

## Notes

Supplement. **eTable 1.** Implementation Dates and Years Among States With an Opioid Prescribing Cap Law Between 2017 and 2019 **eTable 2.** Prescribing Cap Law Provisions Among States With an Opioid Prescribing Cap Law Between 2017 and 2019 **eTable 3.** Subgroup Analysis of Participants Aged 0 to 5 Years **eTable 4.** Subgroup Analysis of Participants Aged 6 to 11 Years **eTable 5.** Subgroup Analysis of Participants Aged 12 to 17 Years **eTable 6.** 2017 Cohort–Specific Associations **eTable 7.** 2018 Cohort–Specific Associations **eTable 8.** 2019 Cohort–Specific Associations **eTable 9.** Adjusted Model **eTable 10.** Prescribing Cap Law Subgroup Analysis of Laws Limiting Duration of Opioid Prescriptions **eTable 11.** Prescribing Cap Law Subgroup Analysis of Laws Limiting Duration and Dose of Opioid Prescriptions **eTable 12.** Prescribing Cap Law Subgroup Analysis of Laws Applying Only to Initial Prescriptions **eTable 13.** Prescribing Cap Law Subgroup Analysis of Laws Not Limited to Initial Prescriptions **eTable 14.** Prescribing Cap Law Subgroup Analysis of Laws That Include a Professional Judgment Exemption **eTable 15.** Prescribing Cap Law Subgroup Analysis of Laws That Do Not Include a Professional Judgment Exemption **eTable 16.** Prescribing Cap Law Subgroup Analysis of Laws That Include a Surgical Pain Exemption **eTable 17.** Prescribing Cap Law Subgroup Analysis of Laws That Do Not Include a Surgical Pain Exemption **eTable 18.** Overall Sample Characteristics **eTable 19.** Treatment State Characteristics **eTable 20.** Control Sample Characteristics
