# Supplementary material for: Association Between State Opioid Prescribing Cap Laws and Receipt of Opioid Prescriptions Among Children and Adolescents
Source: JAMA Health Forum. 2022 Aug 5;3(8):e222461. doi: 10.1001/jamahealthforum.2022.2461 (PMC9356320; doi:10.1001/jamahealthforum.2022.2461)
Supplement: Supplement. — eTable 1. Implementation Dates and Years Among States With an Opioid Prescribing Cap Law Between 2017 and 2019 eTable 2. Prescribing Cap Law Provisions Among States With an Opioid Prescribing Cap Law Between 2017 and 2019 eTable 3. Subgroup Analysis of Participants Aged 0 to 5 Years eTable 4. Subgroup Analysis of Participants Aged 6 to 11 Years eTable 5. Subgroup Analysis of Participants Aged 12 to 17 Years eTable 6. 2017 Cohort–Specific Associations eTable 7. 2018 Cohort–Specific Associations eTable 8. 2019 Cohort–Specific Associations eTable 9. Adjusted Model eTable 10. Prescribing Cap Law Subgroup Analysis of Laws Limiting Duration of Opioid Prescriptions eTable 11. Prescribing Cap Law Subgroup Analysis of Laws Limiting Duration and Dose of Opioid Prescriptions eTable 12. Prescribing Cap Law Subgroup Analysis of Laws Applying Only to Initial Prescriptions eTable 13. Prescribing Cap Law Subgroup Analysis of Laws Not Limited to Initial Prescriptions eTable 14. Prescribing Cap Law Subgroup Analysis of Laws That Include a Professional Judgment Exemption eTable 15. Prescribing Cap Law Subgroup Analysis of Laws That Do Not Include a Professional Judgment Exemption eTable 16. Prescribing Cap Law Subgroup Analysis of Laws That Include a Surgical Pain Exemption eTable 17. Prescribing Cap Law Subgroup Analysis of Laws That Do Not Include a Surgical Pain Exemption eTable 18. Overall Sample Characteristics eTable 19. Treatment State Characteristics eTable 20. Control Sample Characteristics [file jamahealthforum-e222461-s001.pdf]

Supplementary Online Content

Stone EM, Tormohlen KN, McCourt AD, et al. Association between state opioid prescribing cap laws and receipt of opioid prescriptions among children and adolescents. *JAMA Health Forum*. 2022;3(8):e222461. doi:10.1001/jamahealthforum.2022.2461

eTable 1. Implementation Dates and Years Among States With an Opioid Prescribing Cap Law Between 2017 and 2019

eTable 2. Prescribing Cap Law Provisions Among States With an Opioid Prescribing Cap Law Between 2017 and 2019

eTable 3. Subgroup Analysis of Participants Aged 0 to 5 Years

eTable 4. Subgroup Analysis of Participants Aged 6 to 11 Years

eTable 5. Subgroup Analysis of Participants Aged 12 to 17 Years

eTable 6. 2017 Cohort–Specific Associations

eTable 7. 2018 Cohort–Specific Associations

eTable 8. 2019 Cohort–Specific Associations

eTable 9. Adjusted Model

eTable 10. Prescribing Cap Law Subgroup Analysis of Laws Limiting Duration of Opioid Prescriptions

eTable 11. Prescribing Cap Law Subgroup Analysis of Laws Limiting Duration and Dose of Opioid Prescriptions

eTable 12. Prescribing Cap Law Subgroup Analysis of Laws Applying Only to Initial Prescriptions

eTable 13. Prescribing Cap Law Subgroup Analysis of Laws Not Limited to Initial Prescriptions

eTable 14. Prescribing Cap Law Subgroup Analysis of Laws That Include a Professional Judgment Exemption

eTable 15. Prescribing Cap Law Subgroup Analysis of Laws That Do Not Include a Professional Judgment Exemption

eTable 16. Prescribing Cap Law Subgroup Analysis of Laws That Include a Surgical Pain Exemption

eTable 17. Prescribing Cap Law Subgroup Analysis of Laws That Do Not Include a Surgical Pain Exemption

eTable 18. Overall Sample Characteristics

eTable 19. Treatment State Characteristics

eTable 20. Control Sample Characteristics

This supplementary material has been provided by the authors to give readers additional information about their work.

© 2022 Stone EM et al. *JAMA Health Forum.*

eTable 1. Implementation Dates and Years Among States With an Opioid Prescribing Cap Law Between 2017 and 2019

The implementation date below depicts the day the law was implemented. The year of implementation depicts how year was defined in this study. We defined year of implementation as the year in which a state had an opioid prescribing cap law in place for at least 6 months of that year (e.g., a state that implemented an opioid prescribing cap law on March 1, 2017 would have an implementation year of 2017; a state that implemented a law on September 1, 2017 would have an implementation year of 2018).

State	Implementation Date	Year cohort
**CT**	7/1/16	2017
**NY**	7/22/16	2017
**ME**	1/1/17	2017
**NH**	1/1/17	2017
**PA**	2/4/17	2017
**VA**	3/15/17	2017
**RI**	3/22/17	2017
**DE**	4/1/17	2017
**UT**	5/9/17	2017
**NJ**	5/16/17	2017
**MD**	5/25/17	2017
**KY**	6/29/17	2017
**HI**	7/1/17	2018
**IN**	7/1/17	2018
**VT**	7/1/17	2018
**AK**	7/26/17	2018
**LA**	8/1/17	2018
**OH**	8/31/17	2018
**NC**	1/1/18	2018
**NV**	1/1/18	2018
**AZ**	4/26/18	2018
**SC**	5/15/18	2018
**CO**	5/21/18	2018
**WV**	6/7/18	2018
**FL**	7/1/18	2019
**MI**	7/1/18	2019
**TN**	7/1/18	2019
**NE**	7/19/18	2019

© 2022 Stone EM et al. *JAMA Health Forum.*

**AR**	8/15/18	2019
**MO**	8/28/18	2019
**MS**	10/28/18	2019
**OK**	11/1/18	2019
**WA**	1/1/19	2019
**Excluded***	IL, MA	
**Comparison****	AL, CA, GA, IA, ID, KS, MN, MT, ND, NM, OR, SD, TX, WI, WY, DC	

* Law enacted prior to 7/1/2016.
** Had not adopted a prescribing cap law by 7/1/2019. Four states (MN, MT, TX, WY) adopted a law in late 2019. The remaining states and D.C. did not have an eligible law in place by 12/31/19.

© 2022 Stone EM et al. *JAMA Health Forum.*

eTable 2. Prescribing Cap Law Provisions Among States With an Opioid Prescribing Cap Law Between 2017 and 2019

State	Initial effective date	Differs from adult provisions	Limits			Exemptions			
			Day supply limit	Dosage limit	Initial rx only	Professional judgment	Cancer treatment	Surgical pain	Palliative care
AK	7/26/2017	Yes	7	-	No	Yes	Yes	No	Yes
AZ	8/15/2018	No	5	90 MME/day	Yes	Yes	Yes	Yes[a]	Yes
AR	4/26/2018	No	7	Lowest effective	Yes	Yes	Yes	No	Yes
CO	5/21/2018	No	7	-	No	Yes	Yes	Yes	Yes
CT	7/1/2016	Yes	5	-	No	Yes	Yes	No	Yes
DE	4/1/2017	Yes	7	-	No	Yes	Yes	No	Yes
FL	7/1/2018	No	3	-	No	Yes	Yes	No	Yes
HI	7/1/2017	No	7	-	Yes	No	Yes	Yes	Yes
IN	7/1/2017	Yes	7	-	No	Yes	Yes	No	Yes
KY	6/29/2017	No	3	-	No	Yes	Yes	Yes	Yes[b]
LA	8/1/2017	Yes	7	-	No	Yes	Yes	No	Yes
MD	5/25/2017	No	No greater than needed	Lowest effective	No	No	Yes	No	Yes
ME	1/1/2017	No	7 (acute); 30 (chronic)	100 MME/day[c]	No	No	Yes	Yes	Yes
MI	7/1/2018	No	7	-	No	No	No	No	No
MO	8/28/2018	No	7	-	Yes	Yes	Yes	No	Yes
MS	10/28/2018	No	10	Lowest effective	No	Yes	No	No	No
NC	1/1/2018	No	5	-	Yes	No	Yes	Yes	Yes
NE	7/19/2018	Yes	7	-	No	Yes	Yes	No	Yes
NH	1/1/2017	No	7 (ED, urgent care, walk-in clinic)	Lowest effective	No	Yes	Yes	No	No
NJ	5/16/2017	No	5	Lowest effective	Yes	No	Yes	No	Yes
NV	1/1/2018	No	14	90 MME/day	Yes	Yes[d]	Yes[d]	No	Yes[d]
NY	7/22/2016	No	7	-	Yes	No	Yes	No	Yes
OH	8/31/2017	Yes	5	30 MME/day (acute); 120 MME/day	Yes	Yes	Yes	Yes	Yes
OK	11/1/2018	Yes	7	Lowest effective	Yes	No	Yes	Yes[e]	Yes
PA	2/4/2017	Yes	7	-	No	Yes	Yes	No	Yes
RI	3/22/2017	No	20 doses	30 MME/day	Yes	No	Yes	No	Yes
SC	5/15/2018	Yes	7	-	Yes	Yes	Yes	Yes	Yes
TN	7/1/2018	No	Varies	Varies	No	Yes	Yes	No	Yes[f]
UT	5/9/2017	Yes	7	-	No	No	No	Yes	No
VA	3/15/2017	No	7	-	No	Yes	Yes	Yes	Yes
VT	7/1/2017	Yes	3	24 MME/day	Yes	Yes	Yes	Yes	No
WA	1/1/2019	No	7 (acute); 14 (other)	-	No	Yes	Yes	No	Yes
WV	6/7/2018	No	3, 4, 7	Lowest effective	Yes	No	Yes	Yes	Yes
Total	33	12	33	14	14	22	30	13	28

Note: All provisions listed above represent the current state of the law (as of 12/31/2019). For a small number of states, changes were made after the initial effective date. This is denoted by a superscript.

© 2022 Stone EM et al. *JAMA Health Forum.*

[a] Yes as of 8/3/2018
[b] Yes as of 6/27/2019
[C] Decreased from 300 MME per day to 100 MME per day as of 7/1/2017
[d] Yes as of 6/3/2019
[e] Yes as of 5/21/2019
[f] Yes as of 4/9/2019

© 2022 Stone EM et al. *JAMA Health Forum.*

eTable 3. Subgroup Analysis of Participants Aged 0 to 5 Years

Results presented here are from analyses that were limited to individuals aged 0-5.

Year pre/post law implementation	Estimated effect (ATT)	95% confidence interval
Proportion of minors with receipt of at least one opioid prescription, per year		
3 years pre-law	-0.002	-0.007, 0.003
2 years pre-law	0.001	-0.001, 0.004
1 year pre-law	0.000	-0.003, 0.003
1 year post-law	-0.001	-0.003, 0.002
Average number of opioid prescriptions per person, per year		
3 years pre-law	-0.389	-0.740, -0.039*
2 years pre-law	0.163	-0.120, 0.447
1 year pre-law	-0.019	-0.324, 0.287
1 year post-law	0.024	-0.306, 0.354
Average MME per day, per person, per year		
3 years pre-law	-3.924	-8.848, 1.000
2 years pre-law	1.518	-3.694, 6.730
1 year pre-law	-1.379	-6.798, 4.040
1 year post-law	-1.050	-4.260, 2.160
Proportion of minors with receipt of opioid prescription with >30 MME per day, per year		
3 years pre-law	0.0001	-0.004, 0.004
2 years pre-law	-0.005	-0.018, 0.008
1 year pre-law	-0.001	-0.019, 0.016
1 year post-law	-0.013	-0.029, 0.003
Proportion of minors with receipt of opioid prescription with >50 MME per day, per year[1]		
3 years pre-law	-	-
2 years pre-law	-	-
1 year pre-law	-	-
1 year post-law	-	-
Proportion of minors with receipt of opioid prescription with >90 MME per day, per year[1]		
3 years pre-law	-	-
2 years pre-law	-	-
1 year pre-law	-	-
1 year post-law	-	-
Average days' supply, per person, per year		
3 years pre-law	-1.403	-3.517, 0.711
2 years pre-law	0.160	-2.074, 2.394
1 year pre-law	0.273	-2.142, 2.687
1 year post-law	-0.639	-3.979, 2.701
Proportion of minors with receipt of opioid prescription with >3 days' supply, per year		
3 years pre-law	-0.220	-0.441, 0.001
2 years pre-law	0.038	-0.186, 0.262
1 year pre-law	0.023	-0.233, 0.279
1 year post-law	-0.063	-0.301, 0.174
Proportion of minors with receipt of opioid prescription with >5 days' supply, per year		
3 years pre-law	-0.104	-0.317, 0.110
2 years pre-law	0.054	-0.169, 0.277
1 year pre-law	-0.015	-0.263, 0.234

© 2022 Stone EM et al. *JAMA Health Forum.*

1 year post-law	-0.050	-0.276, 0.177
Proportion of minors with receipt of opioid prescription with >7 days' supply, per year		
3 years pre-law	0.007	-0.185, 0.199
2 years pre-law	-0.060	-0.257, 0.136
1 year pre-law	0.037	-0.151, 0.225
1 year post-law	-0.083	-0.267, 0.101

* Indicates confidence interval does not cross 0
[1]Outcome rare in this sample; unable to model effect of prescribing cap law

© 2022 Stone EM et al. *JAMA Health Forum.*

eTable 4. Subgroup Analysis of Participants Aged 6 to 11 Years

Results presented here are from analyses that were limited to individuals aged 6-11.

Year pre/post law implementation	Estimated effect (ATT)	95% confidence interval
Proportion of minors with receipt of at least one opioid prescription, per year		
3 years pre-law	-0.003	-0.008, 0.002
2 years pre-law	0.003	-0.002, 0.008
1 year pre-law	-0.001	-0.004, 0.003
1 year post-law	-0.001	-0.003, 0.002
Average number of opioid prescriptions per person, per year		
3 years pre-law	-0.113	-0.384, 0.158
2 years pre-law	0.034	-0.263, 0.331
1 year pre-law	0.042	-0.269, 0.352
1 year post-law	-0.088	-0.347, 0.170
Average MME per day, per person, per year		
3 years pre-law	-13.987	-36.859, 8.884
2 years pre-law	0.896	-5.235, 7.027
1 year pre-law	-1.327	-8.578, 5.923
1 year post-law	0.728	-9.376, 10.832
Proportion of minors with receipt of opioid prescription with >30 MME per day, per year		
3 years pre-law	-0.069	-0.173, 0.035
2 years pre-law	0.002	-0.029, 0.032
1 year pre-law	-0.017	-0.048, 0.014
1 year post-law	0.018	-0.067, 0.103
Proportion of minors with receipt of opioid prescription with >50 MME per day, per year		
3 years pre-law	-0.030	-0.105, 0.046
2 years pre-law	-0.008	-0.027, 0.011
1 year pre-law	-0.002	-0.016, 0.012
1 year post-law	-0.013	-0.027, 0.002
Proportion of minors with receipt of opioid prescription with >90 MME per day, per year		
3 years pre-law	-0.001	-0.002, 0.001
2 years pre-law	-0.005	-0.013, 0.004
1 year pre-law	-0.004	-0.011, 0.003
1 year post-law	-0.003	-0.008, 0.003
Average days' supply, per person, per year		
3 years pre-law	-0.935	-3.534, 1.665
2 years pre-law	-0.316	-2.190, 1.558
1 year pre-law	-0.072	-1.975, 1.831
1 year post-law	-1.192	-2.820, 0.435
Proportion of minors with receipt of opioid prescription with >3 days' supply, per year		
3 years pre-law	-0.128	-0.367, 0.112
2 years pre-law	0.026	-0.244, 0.296
1 year pre-law	-0.038	-0.288, 0.212
1 year post-law	-0.157	-0.345, 0.031
Proportion of minors with receipt of opioid prescription with >5 days' supply, per year		
3 years pre-law	-0.062	-0.287, 0.163
2 years pre-law	0.001	-0.154, 0.156
1 year pre-law	-0.027	-0.225, 0.172

© 2022 Stone EM et al. *JAMA Health Forum.*

1 year post-law	-0.088	-0.254, 0.079
Proportion of minors with receipt of opioid prescription with >7 days' supply, per year		
3 years pre-law	-0.022	-0.218, 0.174
2 years pre-law	-0.073	-0.217, 0.071
1 year pre-law	-0.010	-0.150, 0.130
1 year post-law	-0.046	-0.166, 0.076

* Indicates confidence interval does not cross 0

© 2022 Stone EM et al. *JAMA Health Forum.*

eTable 5. Subgroup Analysis of Participants Aged 12 to 17 Years

Results presented here are from analyses that were limited to individuals aged 12-17.

Year pre/post law implementation	Estimated effect (ATT)	95% confidence interval
Proportion of minors with receipt of at least one opioid prescription, per year		
3 years pre-law	0.012	-0.031, 0.055
2 years pre-law	-0.008	-0.054, 0.039
1 year pre-law	-0.001	-0.012, 0.009
1 year post-law	-0.003	-0.010, 0.004
Average number of opioid prescriptions per person, per year		
3 years pre-law	-0.110	-0.524, 0.304
2 years pre-law	0.166	-0.110, 0.441
1 year pre-law	-0.097	-0.309, 0.114
1 year post-law	0.117	-0.274, 0.508
Average MME per day, per person, per year		
3 years pre-law	-13.172	-46.943, 20.598
2 years pre-law	5.177	-16.820, 27.174
1 year pre-law	-7.350	-26.824, 12.125
1 year post-law	10.887	-35.116, 56.891
Proportion of minors with receipt of opioid prescription with >30 MME per day, per year		
3 years pre-law	-0.050	-0.245, 0.145
2 years pre-law	0.044	-0.135, 0.223
1 year pre-law	-0.047	-0.197, 0.102
1 year post-law	0.127	-0.046, 0.299
Proportion of minors with receipt of opioid prescription with >50 MME per day, per year		
3 years pre-law	0.002	-0.162, 0.166
2 years pre-law	0.063	-0.099, 0.226
1 year pre-law	-0.102	-0.196, -0.008*
1 year post-law	-0.019	-0.123, 0.085
Proportion of minors with receipt of opioid prescription with >90 MME per day, per year		
3 years pre-law	-0.040	-0.107, 0.026
2 years pre-law	-0.032	-0.080, 0.015
1 year pre-law	-0.005	-0.046, 0.036
1 year post-law	0.042	-0.032, 0.116
Average days' supply, per person, per year		
3 years pre-law	-1.178	-3.102, 0.746
2 years pre-law	0.802	-0.454, 2.058
1 year pre-law	-0.186	-1.824, 1.451
1 year post-law	-0.088	-1.582, 1.406
Proportion of minors with receipt of opioid prescription with >3 days' supply, per year		
3 years pre-law	-0.145	-0.341, 0.051
2 years pre-law	0.154	-0.014, 0.322
1 year pre-law	-0.030	-0.197, 0.137
1 year post-law	-0.051	-0.226, 0.123
Proportion of minors with receipt of opioid prescription with >5 days' supply, per year		
3 years pre-law	-0.136	-0.274, 0.003
2 years pre-law	0.067	-0.068, 0.203
1 year pre-law	-0.070	-0.187, 0.047

© 2022 Stone EM et al. *JAMA Health Forum.*

1 year post-law	-0.003	-0.099, 0.094
Proportion of minors with receipt of opioid prescription with >7 days' supply, per year		
3 years pre-law	-0.099	-0.191, -0.007*
2 years pre-law	0.027	-0.024, 0.077
1 year pre-law	-0.027	-0.069, 0.016
1 year post-law	0.021	-0.050, 0.091

* Indicates confidence interval does not cross 0

© 2022 Stone EM et al. *JAMA Health Forum.*

eTable 6. 2017 Cohort–Specific Associations

Year pre/post law implementation	Estimated effect (ATT)	95% confidence interval
Proportion of minors with receipt of at least one opioid prescription, per year		
3 years pre-law	-0.001	-0.009, 0.008
2 years pre-law	0.005	-0.004, 0.014
1 year pre-law	-0.001	-0.011, 0.009
1 year post-law	-0.005	-0.021, 0.011
2 years post-law	0.004	-0.010, 0.017
3 years post-law	0.001	-0.009, 0.012
Average number of opioid prescriptions per person, per year		
3 years pre-law	-0.126	-0.664, 0.412
2 years pre-law	0.277	-0.091, 0.645
1 year pre-law	-0.273	-1.208, 0.661
1 year post-law	0.300	-0.888, 1.487
2 years post-law	0.499	-0.700, 1.697
3 years post-law	0.180	-0.861, 1.220
Average MME per day, per person, per year		
3 years pre-law	-10.322	-40.368, 19.724
2 years pre-law	19.224	-5.308, 43.756
1 year pre-law	-52.396	-200.133, 95.341
1 year post-law	33.408	-109.659, 176.474
2 years post-law	42.213	-102.281, 186.707
3 years post-law	34.324	-114.821, 183.469
Proportion of minors with receipt of opioid prescription with >30 MME per day, per year		
3 years pre-law	-0.194	-0.590, 0.202
2 years pre-law	0.099	-0.161, 0.359
1 year pre-law	-0.093	-0.553, 0.367
1 year post-law	0.152	-0.296, 0.600
2 years post-law	0.175	-0.271, 0.621
3 years post-law	0.095	-0.266, 0.457
Proportion of minors with receipt of opioid prescription with >50 MME per day, per year		
3 years pre-law	0.024	-0.303, 0.350
2 years pre-law	0.143	0.022, 0.264*
1 year pre-law	-0.166	-0.408, 0.075
1 year post-law	0.043	-0.225, 0.311
2 years post-law	0.018	-0.213, 0.249
3 years post-law	0.044	-0.209, 0.297
Proportion of minors with receipt of opioid prescription with >90 MME per day, per year		
3 years pre-law	-0.014	-0.045, 0.018
2 years pre-law	-0.005	-0.043, 0.033
1 year pre-law	-0.120	-0.318, 0.078
1 year post-law	0.083	-0.130, 0.296
2 years post-law	0.124	-0.074, 0.321
3 years post-law	0.120	-0.075, 0.315
Average days' supply, per person, per year		
3 years pre-law	-2.008	-7.376, 3.360
2 years pre-law	0.962	-1.102, 3.025

© 2022 Stone EM et al. *JAMA Health Forum.*

1 year pre-law	1.054	-3.345, 5.452
1 year post-law	-1.480	-6.166, 3.206
2 years post-law	0.563	-3.700, 4.827
3 years post-law	-1.548	-6.194, 3.099
Proportion of minors with receipt of opioid prescription with >3 days' supply, per year		
3 years pre-law	-0.262	-0.699, 0.176
2 years pre-law	0.355	-0.051, 0.761
1 year pre-law	-0.002	-0.328, 0.323
1 year post-law	-0.161	-0.582, 0.260
2 years post-law	-0.064	-0.625, 0.497
3 years post-law	-0.203	-0.603, 0.197
Proportion of minors with receipt of opioid prescription with >5 days' supply, per year		
3 years pre-law	-0.139	-0.454, 0.177
2 years pre-law	0.045	-0.132, 0.221
1 year pre-law	-0.071	-0.390, 0.249
1 year post-law	-0.051	-0.396, 0.294
2 years post-law	0.009	-0.299, 0.318
3 years post-law	-0.011	-0.321, 0.300
Proportion of minors with receipt of opioid prescription with >7 days' supply, per year		
3 years pre-law	-0.108	-0.367, 0.152
2 years pre-law	0.015	-0.108, 0.138
1 year pre-law	-0.002	-0.226, 0.222
1 year post-law	-0.081	-0.346, 0.183
2 years post-law	-0.025	-0.248, 0.198
3 years post-law	-0.044	-0.286, 0.197

* Indicates confidence interval does not cross 0

© 2022 Stone EM et al. *JAMA Health Forum.*

eTable 7. 2018 Cohort–Specific Associations

Year pre/post law implementation	Estimated effect (ATT)	95% confidence interval
Proportion of minors with receipt of at least one opioid prescription, per year		
4 years pre-law	0.005	-0.004, 0.014
3 years pre-law	0.001	-0.019, 0.021
2 years pre-law	-0.003	-0.021, 0.015
1 year pre-law	-0.004	-0.021, 0.014
1 year post-law	0.003	-0.013, 0.019
2 years post-law	0.004	-0.013, 0.022
Average number of opioid prescriptions per person, per year		
4 years pre-law	0.111	-0.454, 0.676
3 years pre-law	0.119	-0.521, 0.758
2 years pre-law	-0.415	-1.395, 0.565
1 year pre-law	0.299	-0.878, 1.476
1 year post-law	0.092	-0.277, 0.462
2 years post-law	-0.063	-0.473, 0.347
Average MME per day, per person, per year		
4 years pre-law	7.339	-10.287, 24.966
3 years pre-law	3.743	-19.670, 27.156
2 years pre-law	-48.359	-198.510, 101.791
1 year pre-law	43.564	-100.104, 187.233
1 year post-law	3.657	-13.620, 20.934
2 years post-law	-3.535	-19.397, 12.327
Proportion of minors with receipt of opioid prescription with >30 MME per day, per year		
4 years pre-law	-0.021	-0.332, 0.291
3 years pre-law	0.082	-0.314, 0.478
2 years pre-law	-0.182	-0.657, 0.294
1 year pre-law	0.088	-0.313, 0.489
1 year post-law	0.190	-0.147, 0.528
2 years post-law	0.125	-0.148, 0.398
Proportion of minors with receipt of opioid prescription with >50 MME per day, per year		
4 years pre-law	0.090	-0.152, 0.332
3 years pre-law	0.051	-0.130, 0.232
2 years pre-law	-0.138	-0.418, 0.143
1 year pre-law	0.070	-0.187, 0.327
1 year post-law	-0.051	-0.141, 0.039
2 years post-law	-0.015	-0.103, 0.073
Proportion of minors with receipt of opioid prescription with >90 MME per day, per year		
4 years pre-law	-0.016	-0.077, 0.046
3 years pre-law	-0.032	-0.079, 0.016
2 years pre-law	-0.121	-0.314, 0.073
1 year pre-law	0.090	-0.119, 0.299
1 year post-law	0.034	-0.010, 0.078
2 years post-law	0.034	-0.011, 0.079
Average days' supply, per person, per year		
4 years pre-law	0.009	-3.267, 3.286
3 years pre-law	-0.012	-3.167, 3.144

© 2022 Stone EM et al. *JAMA Health Forum.*

2 years pre-law	-0.454	-4.279, 3.371
1 year pre-law	-0.212	-3.820, 3.396
1 year post-law	0.873	-1.262, 3.007
2 years post-law	-0.618	-2.838, 1.602
Proportion of minors with receipt of opioid prescription with >3 days' supply, per year		
4 years pre-law	0.059	-0.288, 0.405
3 years pre-law	0.029	-0.332, 0.390
2 years pre-law	0.067	-0.378, 0.512
1 year pre-law	-0.040	-0.567, 0.487
1 year post-law	0.040	-0.337, 0.417
2 years post-law	-0.141	-0.430, 0.147
Proportion of minors with receipt of opioid prescription with >5 days' supply, per year		
4 years pre-law	0.060	-0.142, 0.262
3 years pre-law	-0.052	-0.249, 0.146
2 years pre-law	0.005	-0.410, 0.420
1 year pre-law	-0.014	-0.363, 0.335
1 year post-law	-0.016	-0.174, 0.142
2 years post-law	-0.060	-0.180, 0.060
Proportion of minors with receipt of opioid prescription with >7 days' supply, per year		
4 years pre-law	0.028	-0.151, 0.207
3 years pre-law	-0.057	-0.195, 0.081
2 years pre-law	-0.099	-0.299, 0.102
1 year pre-law	0.051	-0.155, 0.257
1 year post-law	0.039	-0.032, 0.110
2 years post-law	0.002	-0.084, 0.089

* Indicates confidence interval does not cross 0

© 2022 Stone EM et al. *JAMA Health Forum.*

eTable 8. 2019 Cohort–Specific Associations

Year pre/post law implementation	Estimated effect (ATT)	95% confidence interval
Proportion of minors with receipt of at least one opioid prescription, per year		
5 years pre-law	0.005	-0.006, 0.016
4 years pre-law	-0.003	-0.015, 0.009
3 years pre-law	-0.004	-0.014, 0.007
2 years pre-law	0.004	-0.016, 0.024
1 year pre-law	0.003	-0.013, 0.018
1 year post-law	-0.002	-0.015, 0.012
Average number of opioid prescriptions per person, per year		
5 years pre-law	-0.063	-0.536, 0.411
4 years pre-law	0.074	-0.237, 0.385
3 years pre-law	-0.387	-1.328, 0.555
2 years pre-law	0.395	-0.796, 1.587
1 year pre-law	-0.029	-0.301, 0.242
1 year post-law	-0.239	-0.592, 0.115
Average MME per day, per person, per year		
5 years pre-law	0.343	-16.388, 17.075
4 years pre-law	8.948	-8.670, 26.565
3 years pre-law	-46.225	-194.445, 101.995
2 years pre-law	43.515	-99.918, 186.949
1 year pre-law	0.695	-15.331, 16.721
1 year post-law	-8.632	-21.148, 3.883
Proportion of minors with receipt of opioid prescription with >30 MME per day, per year		
5 years pre-law	-0.023	-0.336, 0.290
4 years pre-law	0.015	-0.228, 0.259
3 years pre-law	0.035	-0.457, 0.526
2 years pre-law	0.058	-0.487, 0.603
1 year pre-law	-0.033	-0.350, 0.285
1 year post-law	-0.040	-0.274, 0.193
Proportion of minors with receipt of opioid prescription with >50 MME per day, per year		
5 years pre-law	0.048	-0.186, 0.282
4 years pre-law	0.131	-0.018, 0.281
3 years pre-law	-0.130	-0.447, 0.187
2 years pre-law	0.116	-0.301, 0.532
1 year pre-law	-0.097	-0.242, 0.047
1 year post-law	-0.013	-0.132, 0.105
Proportion of minors with receipt of opioid prescription with >90 MME per day, per year		
5 years pre-law	-0.035	-0.108, 0.038
4 years pre-law	0.001	-0.040, 0.042
3 years pre-law	-0.070	-0.299, 0.158
2 years pre-law	0.044	-0.190, 0.277
1 year pre-law	0.029	-0.026, 0.084
1 year post-law	0.001	-0.023, 0.025
Average days' supply, per person, per year		
5 years pre-law	-2.505	-8.070, 3.061
4 years pre-law	0.528	-1.621, 2.678

© 2022 Stone EM et al. *JAMA Health Forum.*

3 years pre-law	-0.230	-4.309, 3.849
2 years pre-law	-0.239	-4.569, 4.091
1 year pre-law	-0.236	-1.684, 1.213
1 year post-law	-1.150	-2.574, 0.273
Proportion of minors with receipt of opioid prescription with >3 days' supply, per year		
5 years pre-law	-0.269	-0.721, 0.182
4 years pre-law	0.365	0.027, 0.704*
3 years pre-law	-0.062	-0.422, 0.299
2 years pre-law	-0.176	-0.648, 0.297
1 year pre-law	0.025	-0.282, 0.332
1 year post-law	-0.133	-0.425, 0.158
Proportion of minors with receipt of opioid prescription with >5 days' supply, per year		
5 years pre-law	0.015	-0.304, 0.333
4 years pre-law	0.082	-0.153, 0.317
3 years pre-law	-0.117	-0.576, 0.342
2 years pre-law	0.009	-0.459, 0.476
1 year pre-law	-0.061	-0.199, 0.078
1 year post-law	-0.052	-0.207, 0.102
Proportion of minors with receipt of opioid prescription with >7 days' supply, per year		
5 years pre-law	0.005	-0.213, 0.224
4 years pre-law	-0.007	-0.194, 0.179
3 years pre-law	-0.090	-0.421, 0.242
2 years pre-law	0.037	-0.333, 0.407
1 year pre-law	-0.069	-0.165, 0.028
1 year post-law	-0.008	-0.079, 0.063

* Indicates confidence interval does not cross 0

© 2022 Stone EM et al. *JAMA Health Forum.*

eTable 9. Adjusted Model

These tables present results from balanced models adjusted for sex, age, and proportion with any mental illness or substance use disorder diagnosis in the pre-law period (2013-2016).

Year pre/post law implementation	Estimated effect (ATT)	95% confidence interval
Proportion of minors with receipt of at least one opioid prescription, per year		
3 years pre-law	0.004	-0.006, 0.014
2 years pre-law	0.004	-0.006, 0.014
1 year pre-law	-0.001	-0.014, 0.011
1 year post-law	-0.003	-0.017, 0.011
Average number of opioid prescriptions per person, per year		
3 years pre-law	-0.203	-0.655, 0.249
2 years pre-law	-0.494	-1.613, 0.626
1 year pre-law	-0.114	-1.381, 1.153
1 year post-law	0.134	-0.408, 0.676
Average MME per day, per person, per year		
3 years pre-law	-27.573	-89.185, 34.040
2 years pre-law	-34.333	-117.965, 49.298
1 year pre-law	-26.559	-107.689, 54.570
1 year post-law	20.992	-16.356, 58.340
Proportion of minors with receipt of opioid prescription with >30 MME per day, per year		
3 years pre-law	-0.195	-0.539, 0.149
2 years pre-law	-0.207	-0.553, 0.138
1 year pre-law	-0.195	-0.491, 0.101
1 year post-law	0.231	-0.122, 0.583
Proportion of minors with receipt of opioid prescription with >50 MME per day, per year		
3 years pre-law	-0.124	-0.308, 0.061
2 years pre-law	-0.176	-0.483, 0.131
1 year pre-law	-0.070	-0.294, 0.155
1 year post-law	0.049	-0.072, 0.170
Proportion of minors with receipt of opioid prescription with >90 MME per day, per year		
3 years pre-law	-0.134	-0.239, -0.028*
2 years pre-law	-0.239	-0.469, -0.010*
1 year pre-law	-0.033	-0.240, 0.173
1 year post-law	0.013	-0.087, 0.113
Average days' supply, per person, per year		
3 years pre-law	-1.074	-3.169, 1.021
2 years pre-law	-1.813	-5.793, 2.167
1 year pre-law	0.352	-3.760, 4.464
1 year post-law	-0.065	-3.814, 3.683
Proportion of minors with receipt of opioid prescription with >3 days' supply, per year		
3 years pre-law	-0.039	-0.353, 0.275
2 years pre-law	-0.029	-0.529, 0.471
1 year pre-law	-0.032	-0.359, 0.294
1 year post-law	0.062	-0.284, 0.408
Proportion of minors with receipt of opioid prescription with >5 days' supply, per year		
3 years pre-law	-0.178	-0.339, -0.016*
2 years pre-law	-0.201	-0.610, 0.207

© 2022 Stone EM et al. *JAMA Health Forum.*

1 year pre-law	-0.081	-0.301, 0.139
1 year post-law	-0.130	-0.334, 0.074
Proportion of minors with receipt of opioid prescription with >7 days' supply, per year		
3 years pre-law	-0.092	-0.228, 0.044
2 years pre-law	-0.186	-0.423, 0.051
1 year pre-law	-0.042	-0.251, 0.167
1 year post-law	-0.111	-0.287, 0.065

* Indicates confidence interval does not cross 0

© 2022 Stone EM et al. *JAMA Health Forum.*

eTable 10. Prescribing Cap Law Subgroup Analysis of Laws Limiting Duration of Opioid Prescriptions

Results presented here are from analyses that were limited to the 19 states (AK, CO, CT, DE, FL, HI, IN, KY, LA, MI, MO, NE, NC, NY, PA, SC, UT, VA, WA) with opioid prescribing cap laws that limit day supply of opioid prescriptions. These state laws do not include a dosage limit.

Year pre/post law implementation	Estimated effect (ATT)	95% confidence interval
Proportion of minors with receipt of at least one opioid prescription, per year		
3 years pre-law	0.001	-0.009, 0.010
2 years pre-law	-0.001	-0.010, 0.009
1 year pre-law	0.002	-0.003, 0.006
1 year post-law	-0.002	-0.006, 0.002
Average number of opioid prescriptions per person, per year		
3 years pre-law	-0.085	-0.596, 0.427
2 years pre-law	0.061	-0.335, 0.458
1 year pre-law	0.050	-0.165, 0.265
1 year post-law	0.066	-0.333, 0.464
Average MME per day, per person, per year		
3 years pre-law	-12.375	-43.073, 18.324
2 years pre-law	-0.965	-25.117, 23.186
1 year pre-law	3.517	-16.131, 23.164
1 year post-law	9.219	-34.923, 53.360
Proportion of minors with receipt of opioid prescription with >30 MME per day, per year		
3 years pre-law	-0.089	-0.348, 0.170
2 years pre-law	0.035	-0.175, 0.245
1 year pre-law	0.007	-0.149, 0.163
1 year post-law	0.094	-0.104, 0.293
Proportion of minors with receipt of opioid prescription with >50 MME per day, per year		
3 years pre-law	-0.043	-0.200, 0.114
2 years pre-law	0.061	-0.046, 0.168
1 year pre-law	-0.053	-0.144, 0.039
1 year post-law	-0.017	-0.115, 0.081
Proportion of minors with receipt of opioid prescription with >90 MME per day, per year		
3 years pre-law	-0.046	-0.102, 0.010
2 years pre-law	-0.016	-0.070, 0.039
1 year pre-law	-0.011	-0.067, 0.046
1 year post-law	0.044	-0.030, 0.117
Average days’ supply, per person, per year		
3 years pre-law	-0.759	-3.737, 2.219
2 years pre-law	0.140	-1.498, 1.779
1 year pre-law	0.419	-1.282, 2.119
1 year post-law	-0.682	-2.677, 1.313
Proportion of minors with receipt of opioid prescription with >3 days’ supply, per year		
3 years pre-law	-0.096	-0.327, 0.135
2 years pre-law	0.112	-0.117, 0.341
1 year pre-law	0.038	-0.103, 0.179
1 year post-law	-0.132	-0.310, 0.045
Proportion of minors with receipt of opioid prescription with >5 days’ supply, per year		
3 years pre-law	-0.132	-0.337, 0.073

© 2022 Stone EM et al. *JAMA Health Forum.*

2 years pre-law	0.061	-0.120, 0.241
1 year pre-law	-0.036	-0.187, 0.116
1 year post-law	-0.027	-0.198, 0.144
Proportion of minors with receipt of opioid prescription with >7 days' supply, per year		
3 years pre-law	-0.040	-0.199, 0.119
2 years pre-law	-0.060	-0.199, 0.078
1 year pre-law	0.034	-0.064, 0.132
1 year post-law	-0.034	-0.168, 0.100

* Indicates confidence interval does not cross 0

© 2022 Stone EM et al. *JAMA Health Forum.*

eTable 11. Prescribing Cap Law Subgroup Analysis of Laws Limiting Duration and Dose of Opioid Prescriptions

Results presented here are from analyses that were limited to the 14 states (AZ, AR, MD, ME, MS, NH, NJ, NV, OH, OK, RI, TN, VT, WV) with opioid prescribing cap laws that limit both day supply and dosage of opioid prescriptions.

Year pre/post law implementation	Estimated effect (ATT)	95% confidence interval
Proportion of minors with receipt of at least one opioid prescription, per year		
3 years pre-law	-0.003	-0.007, 0.002
2 years pre-law	0.005	-0.003, 0.013
1 year pre-law	-0.004	-0.010, 0.003
1 year post-law	-0.001	-0.007, 0.006
Average number of opioid prescriptions per person, per year		
3 years pre-law	-0.144	-0.506, 0.219
2 years pre-law	0.058	-0.232, 0.347
1 year pre-law	-0.061	-0.368, 0.246
1 year post-law	0.086	-0.312, 0.483
Average MME per day, per person, per year		
3 years pre-law	-18.729	-55.025, 17.567
2 years pre-law	4.799	-21.885, 31.484
1 year pre-law	-12.086	-46.071, 21.900
1 year post-law	13.532	-29.968, 57.032
Proportion of minors with receipt of opioid prescription with >30 MME per day, per year		
3 years pre-law	0.046	-0.125, 0.216
2 years pre-law	-0.079	-0.296, 0.137
1 year pre-law	-0.033	-0.250, 0.185
1 year post-law	0.136	-0.088, 0.360
Proportion of minors with receipt of opioid prescription with >50 MME per day, per year		
3 years pre-law	0.039	-0.166, 0.244
2 years pre-law	-0.003	-0.228, 0.223
1 year pre-law	-0.072	-0.159, 0.015
1 year post-law	0.005	-0.096, 0.106
Proportion of minors with receipt of opioid prescription with >90 MME per day, per year		
3 years pre-law	-0.022	-0.120, 0.077
2 years pre-law	-0.057	-0.142, 0.027
1 year pre-law	0.006	-0.044, 0.057
1 year post-law	0.042	-0.027, 0.110
Average days' supply, per person, per year		
3 years pre-law	-0.868	-2.379, 0.643
2 years pre-law	0.097	-1.151, 1.345
1 year pre-law	0.047	-2.188, 2.282
1 year post-law	-0.388	-2.405, 1.628
Proportion of minors with receipt of opioid prescription with >3 days' supply, per year		
3 years pre-law	-0.111	-0.320, 0.098
2 years pre-law	0.097	-0.203, 0.397
1 year pre-law	-0.067	-0.325, 0.191
1 year post-law	-0.014	-0.234, 0.207
Proportion of minors with receipt of opioid prescription with >5 days' supply, per year		
3 years pre-law	-0.060	-0.254, 0.134

© 2022 Stone EM et al. *JAMA Health Forum.*

2 years pre-law	-0.034	-0.273, 0.205
1 year pre-law	-0.063	-0.272, 0.145
1 year post-law	-0.055	-0.164, 0.054
Proportion of minors with receipt of opioid prescription with >7 days' supply, per year		
3 years pre-law	-0.145	-0.272, -0.017*
2 years pre-law	0.034	-0.058, 0.126
1 year pre-law	-0.047	-0.108, 0.015
1 year post-law	0.002	-0.072, 0.076

* Indicates confidence interval does not cross 0

© 2022 Stone EM et al. *JAMA Health Forum.*

eTable 12. Prescribing Cap Law Subgroup Analysis of Laws Applying Only to Initial Prescriptions

Results presented here are from analyses that were limited to the 14 states (AZ, AR, HI, MO, NC, NJ, NV, NY, OH, OK, RI, SC, VT, WV) with prescribing cap laws that apply only to initial prescriptions.

Year pre/post law implementation	Estimated effect (ATT)	95% confidence interval
Proportion of minors with receipt of at least one opioid prescription, per year		
3 years pre-law	-0.0004	-0.005, 0.004
2 years pre-law	0.003	-0.005, 0.010
1 year pre-law	-0.002	-0.010, 0.005
1 year post-law	0.001	-0.006, 0.008
Average number of opioid prescriptions per person, per year		
3 years pre-law	-0.172	-0.545, 0.201
2 years pre-law	0.022	-0.330, 0.374
1 year pre-law	0.091	-0.358, 0.540
1 year post-law	0.063	-0.210, 0.336
Average MME per day, per person, per year		
3 years pre-law	-15.243	-42.346, 11.859
2 years pre-law	-9.188	-50.965, 32.588
1 year pre-law	7.089	-33.979, 48.157
1 year post-law	12.063	-14.905, 39.032
Proportion of minors with receipt of opioid prescription with >30 MME per day, per year		
3 years pre-law	-0.077	-0.304, 0.149
2 years pre-law	-0.027	-0.293, 0.240
1 year pre-law	-0.051	-0.307, 0.207
1 year post-law	0.269	0.033, 0.504*
Proportion of minors with receipt of opioid prescription with >50 MME per day, per year		
3 years pre-law	0.048	-0.114, 0.210
2 years pre-law	-0.059	-0.271, 0.154
1 year pre-law	-0.055	-0.169, 0.060
1 year post-law	0.008	-0.058, 0.075
Proportion of minors with receipt of opioid prescription with >90 MME per day, per year		
3 years pre-law	-0.015	-0.108, 0.079
2 years pre-law	-0.086	-0.184, 0.013
1 year pre-law	0.037	-0.050, 0.123
1 year post-law	0.036	-0.011, 0.082
Average days' supply, per person, per year		
3 years pre-law	-1.366	-3.072, 0.340
2 years pre-law	0.545	-1.349, 2.438
1 year pre-law	0.624	-1.990, 3.238
1 year post-law	-0.609	-3.491, 2.273
Proportion of minors with receipt of opioid prescription with >3 days' supply, per year		
3 years pre-law	-0.168	-0.457, 0.121
2 years pre-law	0.189	-0.158, 0.535
1 year pre-law	-0.020	-0.328, 0.289
1 year post-law	-0.031	-0.283, 0.221
Proportion of minors with receipt of opioid prescription with >5 days' supply, per year		
3 years pre-law	-0.072	-0.263, 0.120
2 years pre-law	-0.003	-0.290, 0.285

© 2022 Stone EM et al. *JAMA Health Forum.*

1 year pre-law	-0.008	-0.233, 0.218
1 year post-law	-0.105	-0.293, 0.083
Proportion of minors with receipt of opioid prescription with >7 days' supply, per year		
3 years pre-law	-0.108	-0.234, 0.017
2 years pre-law	-0.024	-0.115, 0.066
1 year pre-law	0.073	-0.083, 0.229
1 year post-law	-0.063	-0.260, 0.133

* Indicates confidence interval does not cross 0

© 2022 Stone EM et al. *JAMA Health Forum.*

eTable 13. Prescribing Cap Law Subgroup Analysis of Laws Not Limited to Initial Prescriptions

Results presented here are from analyses that were limited to the 19 states (AK, CO, CT, DE, FL, IN, KY, LA, MD, ME, MI, MS, NE, NH, PA, TN, UT, VA, WA) with prescribing cap laws that applied to any prescription.

Year pre/post law implementation	Estimated effect (ATT)	95% confidence interval
Proportion of minors with receipt of at least one opioid prescription, per year		
3 years pre-law	-0.001	-0.012, 0.010
2 years pre-law	0.001	-0.009, 0.011
1 year pre-law	0.001	-0.006, 0.007
1 year post-law	-0.003	-0.009, 0.003
Average number of opioid prescriptions per person, per year		
3 years pre-law	-0.067	-0.573, 0.439
2 years pre-law	0.090	-0.286, 0.466
1 year pre-law	-0.054	-0.336, 0.229
1 year post-law	0.074	-0.390, 0.538
Average MME per day, per person, per year		
3 years pre-law	-15.059	-54.460, 24.343
2 years pre-law	9.533	-15.206, 34.272
1 year pre-law	-10.654	-46.396, 25.088
1 year post-law	10.235	-47.439, 67.909
Proportion of minors with receipt of opioid prescription with >30 MME per day, per year		
3 years pre-law	0.001	-0.239, 0.240
2 years pre-law	-0.003	-0.211, 0.205
1 year pre-law	0.024	-0.147, 0.194
1 year post-law	-0.007	-0.180, 0.166
Proportion of minors with receipt of opioid prescription with >50 MME per day, per year		
3 years pre-law	-0.051	-0.221, 0.120
2 years pre-law	0.104	-0.003, 0.211
1 year pre-law	-0.066	-0.161, 0.030
1 year post-law	-0.020	-0.138, 0.099
Proportion of minors with receipt of opioid prescription with >90 MME per day, per year		
3 years pre-law	-0.052	-0.125, 0.022
2 years pre-law	0.006	-0.051, 0.062
1 year pre-law	-0.033	-0.108, 0.041
1 year post-law	0.048	-0.061, 0.158
Average days' supply, per person, per year		
3 years pre-law	-0.403	-3.504, 2.699
2 years pre-law	-0.183	-1.972, 1.607
1 year pre-law	0.025	-1.921, 1.970
1 year post-law	-0.549	-2.418, 1.320
Proportion of minors with receipt of opioid prescription with >3 days' supply, per year		
3 years pre-law	-0.055	-0.267, 0.157
2 years pre-law	0.045	-0.129, 0.219
1 year pre-law	0.008	-0.144, 0.159
1 year post-law	-0.124	-0.307, 0.059
Proportion of minors with receipt of opioid prescription with >5 days' supply, per year		
3 years pre-law	-0.124	-0.330, 0.083
2 years pre-law	0.038	-0.126, 0.202

© 2022 Stone EM et al. *JAMA Health Forum.*

1 year pre-law	-0.076	-0.187, 0.036
1 year post-law	0.009	-0.118, 0.136
Proportion of minors with receipt of opioid prescription with >7 days' supply, per year		
3 years pre-law	-0.067	-0.244, 0.110
2 years pre-law	-0.017	-0.166, 0.132
1 year pre-law	-0.054	-0.126, 0.017
1 year post-law	0.014	-0.073, 0.102

* Indicates confidence interval does not cross 0

© 2022 Stone EM et al. *JAMA Health Forum.*

eTable 14. Prescribing Cap Law Subgroup Analysis of Laws That Include a Professional Judgment Exemption

Results presented here are from analyses that were limited to the 21 states (AK, AZ, AR, CO, CT, DE, FL, IN, KY, LA, MO, MS, NE, NH, NV, OH, PA, TN, VA, VT, WA) with opioid prescribing cap laws that include a professional judgement exemption.

Year pre/post law implementation	Estimated effect (ATT)	95% confidence interval
Proportion of minors with receipt of at least one opioid prescription, per year		
3 years pre-law	-0.001	-0.011, 0.008
2 years pre-law	0.002	-0.007, 0.011
1 year pre-law	-0.001	-0.006, 0.004
1 year post-law	-0.001	-0.005, 0.004
Average number of opioid prescriptions per person, per year		
3 years pre-law	-0.090	-0.549, 0.369
2 years pre-law	0.083	-0.278, 0.444
1 year pre-law	0.015	-0.275, 0.305
1 year post-law	0.002	-0.265, 0.268
Average MME per day, per person, per year		
3 years pre-law	-14.965	-53.728, 23.798
2 years pre-law	-2.782	-25.224, 19.660
1 year pre-law	10.084	-9.654, 29.823
1 year post-law	2.743	-31.389, 36.874
Proportion of minors with receipt of opioid prescription with >30 MME per day, per year		
3 years pre-law	-0.046	-0.276, 0.184
2 years pre-law	-0.027	-0.223, 0.169
1 year pre-law	0.066	-0.066, 0.199
1 year post-law	0.017	-0.108, 0.142
Proportion of minors with receipt of opioid prescription with >50 MME per day, per year		
3 years pre-law	-0.044	-0.200, 0.112
2 years pre-law	0.042	-0.102, 0.186
1 year pre-law	-0.059	-0.140, 0.022
1 year post-law	-0.016	-0.102, 0.070
Proportion of minors with receipt of opioid prescription with >90 MME per day, per year		
3 years pre-law	-0.024	-0.099, 0.052
2 years pre-law	-0.041	-0.106, 0.024
1 year pre-law	0.009	-0.042, 0.059
1 year post-law	0.038	-0.016, 0.093
Average days' supply, per person, per year		
3 years pre-law	-0.452	-3.206, 2.303
2 years pre-law	-0.152	-1.787, 1.484
1 year pre-law	-0.539	-1.596, 0.517
1 year post-law	0.047	-1.067, 1.161
Proportion of minors with receipt of opioid prescription with >3 days' supply, per year		
3 years pre-law	-0.021	-0.226, 0.185
2 years pre-law	0.025	-0.186, 0.236
1 year pre-law	0.006	-0.198, 0.209
1 year post-law	-0.108	-0.249, 0.032
Proportion of minors with receipt of opioid prescription with >5 days' supply, per year		
3 years pre-law	-0.096	-0.278, 0.086

© 2022 Stone EM et al. *JAMA Health Forum.*

2 years pre-law	-0.002	-0.180, 0.175
1 year pre-law	-0.040	-0.173, 0.093
1 year post-law	-0.003	-0.107, 0.101
Proportion of minors with receipt of opioid prescription with >7 days' supply, per year		
3 years pre-law	-0.060	-0.211, 0.091
2 years pre-law	-0.050	-0.183, 0.084
1 year pre-law	-0.037	-0.097, 0.024
1 year post-law	0.039	-0.017, 0.094

* Indicates confidence interval does not cross 0

© 2022 Stone EM et al. *JAMA Health Forum.*

eTable 15. Prescribing Cap Law Subgroup Analysis of Laws That Do Not Include a Professional Judgment Exemption

Results presented here are from analyses that were limited to the 12 states (HI, MD, ME, MI, NC, NJ, NY, OK, RI, SC, UT, WV) with opioid prescribing cap laws that do not include a professional judgement exemption.

Year pre/post law implementation	Estimated effect (ATT)	95% confidence interval
Proportion of minors with receipt of at least one opioid prescription, per year		
3 years pre-law	0.001	-0.004, 0.005
2 years pre-law	0.001	-0.003, 0.005
1 year pre-law	-0.0002	-0.009, 0.009
1 year post-law	-0.002	-0.010, 0.005
Average number of opioid prescriptions per person, per year		
3 years pre-law	-0.148	-0.536, 0.240
2 years pre-law	0.014	-0.176, 0.203
1 year pre-law	-0.017	-0.291, 0.258
1 year post-law	0.216	-0.389, 0.821
Average MME per day, per person, per year		
3 years pre-law	-15.289	-47.232, 16.655
2 years pre-law	9.978	-21.641, 41.597
1 year pre-law	-29.285	-73.575, 15.006
1 year post-law	27.515	-40.615, 95.644
Proportion of minors with receipt of opioid prescription with >30 MME per day, per year		
3 years pre-law	-0.003	-0.249, 0.244
2 years pre-law	0.013	-0.270, 0.295
1 year pre-law	-0.160	-0.457, 0.137
1 year post-law	0.300	-0.020, 0.620
Proportion of minors with receipt of opioid prescription with >50 MME per day, per year		
3 years pre-law	0.063	-0.105, 0.231
2 years pre-law	0.018	-0.179, 0.215
1 year pre-law	-0.067	-0.197, 0.063
1 year post-law	0.012	-0.123, 0.146
Proportion of minors with receipt of opioid prescription with >90 MME per day, per year		
3 years pre-law	-0.060	-0.117, -0.003*
2 years pre-law	-0.018	-0.071, 0.036
1 year pre-law	-0.027	-0.116, 0.061
1 year post-law	0.052	-0.057, 0.161
Average days' supply, per person, per year		
3 years pre-law	-1.504	-3.532, 0.524
2 years pre-law	0.672	-0.785, 2.129
1 year pre-law	1.837	-1.653, 5.326
1 year post-law	-1.744	-5.314, 1.826
Proportion of minors with receipt of opioid prescription with >3 days' supply, per year		
3 years pre-law	-0.265	-0.572, 0.042
2 years pre-law	0.268	-0.075, 0.611
1 year pre-law	-0.029	-0.318, 0.259
1 year post-law	-0.033	-0.350, 0.285
Proportion of minors with receipt of opioid prescription with >5 days' supply, per year		
3 years pre-law	-0.113	-0.335, 0.110

© 2022 Stone EM et al. *JAMA Health Forum.*

2 years pre-law	0.067	-0.160, 0.293
1 year pre-law	-0.061	-0.296, 0.174
1 year post-law	-0.112	-0.324, 0.100
Proportion of minors with receipt of opioid prescription with >7 days' supply, per year		
3 years pre-law	-0.133	-0.309, 0.044
2 years pre-law	0.038	-0.043, 0.119
1 year pre-law	0.069	-0.116, 0.254
1 year post-law	-0.129	-0.358, 0.099

* Indicates confidence interval does not cross 0

© 2022 Stone EM et al. *JAMA Health Forum.*

eTable 16. Prescribing Cap Law Subgroup Analysis of Laws That Include a Surgical Pain Exemption

Results presented here are from analyses that were limited to the 13 states (AZ, CO, HI, KY, ME, NC, OH, OK, SC, UT, VA, VT, WV) with opioid prescribing cap laws that include a surgical pain exemption.

Year pre/post law implementation	Estimated effect (ATT)	95% confidence interval
Proportion of minors with receipt of at least one opioid prescription, per year		
3 years pre-law	-0.002	-0.010, 0.006
2 years pre-law	0.002	-0.003, 0.007
1 year pre-law	-0.003	-0.011, 0.005
1 year post-law	0.004	-0.003, 0.011
Average number of opioid prescriptions per person, per year		
3 years pre-law	-0.031	-0.450, 0.388
2 years pre-law	-0.072	-0.572, 0.428
1 year pre-law	0.017	-0.410, 0.444
1 year post-law	0.240	-0.126, 0.606
Average MME per day, per person, per year		
3 years pre-law	-3.169	-20.849, 14.511
2 years pre-law	-16.110	-76.329, 44.110
1 year pre-law	11.350	-21.251, 43.951
1 year post-law	11.146	-24.059, 46.350
Proportion of minors with receipt of opioid prescription with >30 MME per day, per year		
3 years pre-law	-0.041	-0.260, 0.179
2 years pre-law	0.056	-0.216, 0.328
1 year pre-law	-0.040	-0.227, 0.147
1 year post-law	0.211	-0.022, 0.444
Proportion of minors with receipt of opioid prescription with >50 MME per day, per year		
3 years pre-law	0.048	-0.111, 0.207
2 years pre-law	-0.002	-0.198, 0.194
1 year pre-law	-0.064	-0.185, 0.057
1 year post-law	-0.037	-0.109, 0.036
Proportion of minors with receipt of opioid prescription with >90 MME per day, per year		
3 years pre-law	-0.039	-0.089, 0.012
2 years pre-law	-0.070	-0.191, 0.051
1 year pre-law	0.015	-0.082, 0.113
1 year post-law	0.049	-0.020, 0.118
Average days' supply, per person, per year		
3 years pre-law	-0.293	-2.542, 1.955
2 years pre-law	0.232	-1.819, 2.284
1 year pre-law	-0.751	-2.346, 0.845
1 year post-law	0.874	-0.549, 2.297
Proportion of minors with receipt of opioid prescription with >3 days' supply, per year		
3 years pre-law	0.005	-0.250, 0.260
2 years pre-law	0.150	-0.161, 0.460
1 year pre-law	-0.094	-0.362, 0.174
1 year post-law	0.003	-0.267, 0.273
Proportion of minors with receipt of opioid prescription with >5 days' supply, per year		
3 years pre-law	-0.008	-0.185, 0.169
2 years pre-law	-0.001	-0.274, 0.271

© 2022 Stone EM et al. *JAMA Health Forum.*

1 year pre-law	-0.096	-0.319, 0.126
1 year post-law	0.007	-0.096, 0.111
Proportion of minors with receipt of opioid prescription with >7 days' supply, per year		
3 years pre-law	-0.021	-0.140, 0.098
2 years pre-law	-0.080	-0.193, 0.034
1 year pre-law	0.018	-0.059, 0.095
1 year post-law	0.033	-0.037, 0.102

* Indicates confidence interval does not cross 0

© 2022 Stone EM et al. *JAMA Health Forum.*

eTable 17. Prescribing Cap Law Subgroup Analysis of Laws That Do Not Include a Surgical Pain Exemption

Results presented here are from analyses that were limited to the 20 states (AK, AR, CT, DE, FL, IN, LA, MD, MI, MO, MS, NE, NH, NJ, NV, NY, PA, RI, TN, WA) with opioid prescribing cap laws that do not include a surgical pain exemption.

Year pre/post law implementation	Estimated effect (ATT)	95% confidence interval
Proportion of minors with receipt of at least one opioid prescription, per year		
3 years pre-law	-0.0002	-0.010, 0.010
2 years pre-law	0.002	-0.009, 0.012
1 year pre-law	0.001	-0.006, 0.008
1 year post-law	-0.005	-0.010, 0.000
Average number of opioid prescriptions per person, per year		
3 years pre-law	-0.164	-0.658, 0.330
2 years pre-law	0.148	-0.241, 0.538
1 year pre-law	0.0002	-0.266, 0.266
1 year post-law	-0.040	-0.432, 0.352
Average MME per day, per person, per year		
3 years pre-law	-22.975	-71.664, 25.714
2 years pre-law	13.172	-16.888, 43.231
1 year pre-law	-12.400	-43.812, 19.012
1 year post-law	10.775	-41.864, 63.414
Proportion of minors with receipt of opioid prescription with >30 MME per day, per year		
3 years pre-law	-0.028	-0.269, 0.213
2 years pre-law	-0.057	-0.282, 0.168
1 year pre-law	0.010	-0.218, 0.238
1 year post-law	0.047	-0.148, 0.243
Proportion of minors with receipt of opioid prescription with >50 MME per day, per year		
3 years pre-law	-0.045	-0.236, 0.145
2 years pre-law	0.058	-0.091, 0.208
1 year pre-law	-0.061	-0.151, 0.029
1 year post-law	0.013	-0.092, 0.119
Proportion of minors with receipt of opioid prescription with >90 MME per day, per year		
3 years pre-law	-0.034	-0.130, 0.062
2 years pre-law	-0.009	-0.088, 0.069
1 year pre-law	-0.016	-0.073, 0.042
1 year post-law	0.039	-0.042, 0.119
Average days' supply, per person, per year		
3 years pre-law	-1.144	-3.984, 1.695
2 years pre-law	0.061	-1.671, 1.792
1 year pre-law	0.939	-1.176, 3.054
1 year post-law	-1.515	-3.595, 0.566
Proportion of minors with receipt of opioid prescription with >3 days' supply, per year		
3 years pre-law	-0.173	-0.415, 0.069
2 years pre-law	0.079	-0.184, 0.342
1 year pre-law	0.059	-0.137, 0.255
1 year post-law	-0.148	-0.315, 0.020
Proportion of minors with receipt of opioid prescription with >5 days' supply, per year		
3 years pre-law	-0.163	-0.362, 0.036
2 years pre-law	0.036	-0.156, 0.228

© 2022 Stone EM et al. *JAMA Health Forum.*

1 year pre-law	-0.013	-0.148, 0.122
1 year post-law	-0.072	-0.239, 0.094
Proportion of minors with receipt of opioid prescription with >7 days' supply, per year		
3 years pre-law	-0.126	-0.286, 0.034
2 years pre-law	0.018	-0.123, 0.160
1 year pre-law	-0.013	-0.129, 0.103
1 year post-law	-0.051	-0.186, 0.083

* Indicates confidence interval does not cross 0

© 2022 Stone EM et al. *JAMA Health Forum.*

eTable 18. Overall Sample Characteristics (N = 482 118)

	2013	2014	2015	2016	2017	2018	2019
N (person-years)	98,371	79,629	71,999	78,620	89,559	102,435	233,755
**State demographics**							
Female (%)	49.2	49.3	49.7	48.2	49.7	47.7	48.6
Mean Age (years)	9.6	10.0	10.3	10.6	10.6	10.7	10.1
Age groups (%)							
0-5	22.8	21.6	21.0	17.9	17.3	16.4	19.9
6-11	35.2	35.2	35.7	36.5	37.5	37.9	36.7
12-17	42.0	43.2	43.3	45.7	45.2	45.7	43.4
Any mental illness (%)	6.2	5.9	7.0	6.3	7.9	8.8	10.3
Any substance use disorder (%)	0.1	0.1	0.1	0.1	0.1	0.2	0.1
**Opioid prescriptions**							
Receipt of least one opioid rx (%)	0.9	1.3	1.4	0.7	1.4	1.0	1.7
Among those with at least one opioid rx:							
Number of opioid rx	1.1	1.0	1.0	1.0	0.9	0.9	1.1
Average days' supply	5.0	4.0	4.1	4.4	3.6	3.7	3.3
Average MME/day	33.3	30.6	35.0	43.9	31.6	26.0	29.1
Receipt of opioid rx with: (%)							
>30 MME/day	40.1	38.6	39.3	39.8	40.8	29.2	32.6
>50 MME/day	15.6	11.7	10.9	12.0	10.5	3.1	2.4
>90 MME/day	2.3	1.0	1.3	5.4	1.6	0.4	0.4
Receipt of opioid rx with: (%)							
>3 days' supply	49.5	47.9	42.8	49.8	41.8	40.7	12.0
>5 days' supply	22.2	16.2	20.6	24.0	14.2	11.9	6.2
>7 days' supply	12.9	9.8	9.7	11.6	6.9	3.0	2.8

© 2022 Stone EM et al. *JAMA Health Forum.*

eTable 19. Treatment State Characteristics (n = 278 641)

	2013	2014	2015	2016	2017	2018	2019
N (person-years)	46,016	41,278	39,196	44,239	52,096	57,252	141,397
**State demographics**							
Female (%)	49.4	49.6	50.3	48.0	49.9	47.0	48.5
Mean Age (years)	9.5	10.0	10.3	10.7	10.7	10.7	10.2
Age groups (%)							
0-5	22.2	21.3	20.6	16.6	15.7	14.9	19.5
6-11	35.2	34.9	35.7	36.2	37.5	37.9	36.6
12-17	42.5	43.8	43.7	47.2	46.8	47.2	43.8
Any mental illness (%)	6.2	5.8	7.3	6.3	8.1	9.1	10.6
Any substance use disorder (%)	0.1	0.1	0.1	0.1	0.1	0.2	0.1
**Opioid prescriptions**							
Receipt of least one opioid rx (%)	0.7	1.1	1.3	0.5	1.1	1.0	1.6
Among those with at least one opioid rx:							
Number of opioid rx	1.1	1.0	1.1	0.9	0.9	1.0	1.1
Average days' supply	5.4	4.0	4.2	4.7	3.6	4.0	3.1
Average MME/day	32.7	29.7	37.5	30.4	31.1	27.0	27.5
Receipt of opioid rx with: (%)							
>30 MME/day	39.3	35.0	38.1	35.6	40.1	30.4	31.9
>50 MME/day	13.4	11.4	14.0	10.3	11.2	2.5	2.0
>90 MME/day	3.4	1.3	1.2	1.8	0.5	0.4	0.4
Receipt of opioid rx with: (%)							
>3 days' supply	51.6	45.2	47.9	55.1	43.2	43.6	10.2
>5 days' supply	25.1	18.2	23.4	25.0	14.4	12.1	5.2
>7 days' supply	16.0	12.0	11.4	11.2	6.6	3.1	2.2

© 2022 Stone EM et al. *JAMA Health Forum.*

eTable 20. Control Sample Characteristics (n = 203 477)

	2013	2014	2015	2016	2017	2018	2019
N (person-years)	52,355	38,351	32,803	34,381	37,463	45,183	92,358
**State demographics**							
Female (%)	48.9	48.7	48.6	48.7	49.2	49.2	49.0
Mean Age (years)	9.9	10.0	10.2	10.5	10.4	10.7	10.1
Age groups (%)							
0-5	23.3	22.0	21.5	19.6	19.4	18.3	20.4
6-11	35.3	35.5	35.8	36.8	37.5	37.8	36.8
12-17	41.5	42.5	42.7	43.6	43.0	43.9	42.8
Any mental illness (%)	6.1	5.9	6.3	6.3	7.6	8.0	9.7
Any substance use disorder (%)	0.1	0.1	0.1	0.1	0.2	0.2	0.2
**Opioid prescriptions**							
Receipt of least one opioid rx (%)	1.4	1.5	1.6	1.0	1.9	1.2	2.0
Among those with at least one opioid rx:							
Number of opioid rx	1.1	1.1	1.0	1.2	0.9	0.8	1.1
Average days supply	4.0	4.0	3.7	4.0	3.6	3.1	3.8
Average MME/day	34.7	32.7	29.7	71.9	32.7	23.9	32.3
Receipt of opioid rx with: (%)							
>30 MME/day	41.7	45.9	41.9	48.5	42.3	26.1	34.0
>50 MME/day	20.0	12.5	4.5	15.4	9.0	5.7	3.3
>90 MME/day	0.2	0.2	1.4	12.7	3.9	0.3	0.3
Receipt of opioid rx with: (%)							
>3 days' supply	45.2	53.5	32.3	38.9	39.1	33.8	15.7
>5 days' supply	16.2	11.9	15.0	22.1	13.8	11.4	8.3
>7 days' supply	6.5	5.2	6.3	12.3	7.6	2.6	4.0

© 2022 Stone EM et al. *JAMA Health Forum.*
